# The modern scientific interpretation of ancient wisdom: a review of the phytochemistry and pharmacology of Erzhi Pill and its constituent botanical drugs

**DOI:** 10.3389/fphar.2026.1797126

**Published:** 2026-04-21

**Authors:** Xiaoya Li, Xingyu Liu, Jingyi Jiang, Meirong Fang, Shengchen Wang, Xiaoshuang Mao, Xiwen Li, Cheng Xiao, Wen Jin

**Affiliations:** 1 The Institute of Medicinal Plant Development, Chinese Academy of Medical Sciences & Peking Union Medical College, Beijing, China; 2 State Key Laboratory for Quality Ensurance and Sustainable Use of Dao-di Herbs, Beijing, China; 3 Key Laboratory of Bioactive Substances and Resource Utilization of Chinese Herbal Medicine, Ministry of Education, Beijing, China; 4 Key Laboratory of Efficacy Evaluation of Chinese Medicine against Glycolipid Metabolism Disorder Disease, State Administration of Traditional Chinese Medicine, Beijing, China; 5 Institute of Clinical Medical Sciences, China-Japan Friendship Hospital, Beijing, China

**Keywords:** Ligustri Lucidi Fructus, Ecliptae Herba, Erzhi Pill, phytochemistry, pharmacological activities, further development

## Abstract

Erzhi Pill has been used in traditional Chinese medicine for nearly six centuries. It concurrently delivers nourishing Liver-Kidney Yin (lower) and clears floating deficiency fire (upper) to holistically tackle both the root cause and symptoms of disorders including osteoporosis and menopausal syndrome. Thus, Erzhi Pill has an established reputation as the primary formula for “clearing the upper and tonifying the lower.” While modern clinical and experimental evidence has demonstrated the therapeutic potential of Erzhi Pill, its further development is impeded by several challenges, including simplistic quality control markers, unclear pharmacological mechanisms, and imprecise clinical indications. To support the quality standardization and enhanced clinical use of Erzhi Pill, this review systematically evaluates its constituent botanical drugs and final formula across multiple domains, including the resource distribution of its metabolite constituent botanical drugs, phytochemical profiles, efficacy, mechanisms, and applications, thereby establishing a foundation for further development of Erzhi Pill. Furthermore, we summarized future research directions of Erzhi Pill, which may include exploring integrated quality control markers and pharmacodynamic substances, adopting synchronized screening strategies for active ingredients and targets to elucidate the target network, and clarifying indications and precise medication strategies through evidence-based medical methodologies.

## 1 Introduction

The global acceptance of traditional Chinese medicine (TCM) has been steadily growing, driven by its systematic integration into healthcare systems worldwide (Zhu et al., 2009). The World Health Organization estimated that if traditional medicine can be scientifically validated, it may hold the potential to bridge healthcare access gaps for millions worldwide (News, 2023). Similar to other TCM prescriptions, Erzhi Pill faces challenges in secondary development and global recognition due to its complex metabolites, lack of robust quality control, poorly defined multitarget mechanisms, and ambiguous clinical indications.

Erzhi Pill is a metabolite formula born of ancient wisdom, first documented as “Nvzhen Dan” in *Fu Shou Jing Fang* during the Ming Dynasty (1536 CE) and composed of the fundamental herbal pair Ligustri Lucidi Fructus and Ecliptae Herba. Despite its ancient origins, it continues to be highly relevant today. Centuries of empirical application have confirmed its high safety profile and significant therapeutic efficacy in managing multisystem disorders characterized by Liver–Kidney Yin deficiency, manifested as Xuan Yun Er Ming (vertigo, dizziness, and tinnitus), Yan Gan Bi Zao (dry throat and nose), and Yue Jing Liang Duo (heavy menstruation) (Commission, 2025). Specifically, Erzhi Pill has shown excellent therapeutic efficacy in menopausal syndrome (Yang et al., 2009; Chen et al., 2011), which currently lacks particularly effective treatments and requires long-term medication in clinical practice. Furthermore, modern science has now shed light on the remarkable therapeutic potential of Erzhi Pill in treating liver diseases (e.g., chronic hepatitis B) (Zhao et al., 2019), metabolic bone disease (e.g., osteoporosis) (Cheng et al., 2011; Liang et al., 2018; Qin et al., 2021), and cancer (e.g., breast cancer) (Duan et al., 2021; Zheng and Pan, 2022). Due to its mild nature, Erzhi Pill is a foundational formula that is frequently incorporated into other metabolite prescriptions, such as Er Zhi Tian Gui formula (EZTG). Due to its complex metabolic profile, Erzhi Pill exerts its pharmacological effects through multiple pathways and targets, with the mitigation of oxidative stress serving as a common mechanism across its various indications.

While numerous studies have explored the chemical composition (Jing et al., 2025), pharmacology (Chen et al., 2024), and clinical use (Yang et al., 2009; Chen et al., 2011) of Erzhi Pill, no review has summarized the research on the botany, chemical composition, pharmacology, traditional uses, and toxicity of Erzhi Pill. There is an urgent need for a comprehensive review of Erzhi Pill in order to more deeply understand how the use of this ancient formula based on traditional wisdom translates into verifiable modern effects.

Therefore, this review goes beyond a conventional summary by undertaking a comparative analysis of Erzhi Pill and its constituent botanical drugs, Ligustri Lucidi Fructus and Ecliptae Herba. We juxtaposed their profiles in plant resources, phytochemistry, quality control, pharmacological research, and traditional and modern applications to decipher the scientific rationale underlying their combined efficacy as Erzhi Pill. Ultimately, this work provides strong support for the quality standardization and clinical use of Erzhi Pill and other secondary developments. We propose that multidisciplinary approaches to authenticate botanical drug origins, DNA barcoding to identify counterfeit pills, synergistic screening of bioactive metabolites with efficacy targets, enhanced non-clinical toxicology data, and evidence-based precision medication interventions can comprehensively address the challenges in the continued development and global application of Erzhi Pill.

## 2 Sources and methods

### 2.1 Resources and search strategies

A literature search was performed across electronic databases, including PubMed, Web of Science, and Google Scholar, specialized books, and official websites. The searches included all records available from the inception of each database until October 2025.

To ensure systematic retrieval of sources, a comprehensive search strategy was developed using Boolean operators. For Ligustri Lucidi Fructus, the search string was as follows: (“Glossy Privet Fruit” [Title/Abstract] OR “Ligustri Lucidi Fructus” [Title/Abstract] OR “Ligustrum lucidum” [Title/Abstract]). For Ecliptae Herba, the search string was as follows: (“Ecliptae Herba” [Title/Abstract] OR “Eclipta prostrata” [Title/Abstract] OR “false daisy” [Title/Abstract]). For Erzhi Pill, the search string was as follows: (“Erzhi Wan” [Title/Abstract] OR “Erzhi Pill” [Title/Abstract] OR “Erzhi Formula” [Title/Abstract] OR “Erzhi Granule” [Title/Abstract] OR “EZW” [Title/Abstract] OR “EZP” [Title/Abstract]).

The search was restricted to articles published in English only. No publication status restrictions were imposed. However, retracted articles were excluded during the screening process based on database withdrawal notices and manual verification.

The full text of the potentially relevant articles was then assessed for eligibility. The titles and abstracts of all retrieved records were independently screened by two authors (Xiaoya Li and Xingyu Liu) to identify studies related to the pharmacodynamics and mechanisms of Erzhi Pill and its constituent botanical drugs. Discrepancies were resolved through a discussion or consultation with a third author (Wen Jin).

Only studies reporting core pharmacodynamic effects or mechanisms with statistically significant differences compared with the control group were included; otherwise, the study was excluded during the full-text assessment.

### 2.2 Geographic distribution of Ligustri Lucidi Fructus and Ecliptae Herba

Native areas and those where Ligustri Lucidi Fructus and Ecliptae Herba were introduced were identified using two sources: *Catalogue of Life China: 2024 Annual Checklist* (http://www.sp2000.org.cn/CoLChina) and *Plants of the World Online* (http://www.plantsoftheworldonline.org/). The geographic distribution information from these sources was first converted into standardized taxonomic codes. These codes were subsequently used as input for the SimpleMappr platform (https://www.simplemappr.net/) to produce the distribution maps.

### 2.3 Phytochemical profiling

Plant chemical metabolites were systematically investigated through comprehensive literature mining. Information on the metabolites reported in peer-reviewed studies was initially extracted to generate a dataset of purified metabolites. The dataset was classified into major phytochemical categories (e.g., alkaloids, flavonoids, and terpenoids) based on the structural characteristics and biosynthetic origins of the metabolites. The primary bioactive chemical metabolites were rendered using ChemDraw.

### 2.4 Pharmacological evaluation and mechanistic elucidation

Pharmacodynamic data and therapeutic indications were curated from experimental studies. Molecular targets and signaling pathways described in the literature were rigorously referenced against the Kyoto Encyclopedia of Genes and Genomes, PATHWAY, Reactome, WikiPathways, Lipid Metabolites and Pathways Strategy, and PathBank to standardize the biological pathway nomenclature. Finally, the collected information was organized into tables and visualized using petal and radar charts created with ChiPlot (https://www.chiplot.online/) and network diagrams created with Cytoscape.

## 3 Botany

### 3.1 Taxonomic classification and common names

Ligustri Lucidi Fructus, commonly known as Nvzhenzi and glossy privet fruit, is the desiccated ripe fruit (Commission, 2025) of the shrub or tree (Institute Of Botany, 1996) *Ligustrum lucidum* W.T.Aiton, belonging to the genus *Ligustrum* and family *Oleaceae* (Bánki et al., 2022).

Ecliptae Herba, also called Mohanlian or Yervadetajo, is the dried aerial part (Commission, 2025) of the annual herbaceous plant (Tewtrakul et al., 2011) *Eclipta prostrata* (L.) L., with the synonyms *E. alba* Hassk. and *E. erecta* Hassk (Tewtrakul et al., 2011). and vernacular names false daisy (Abbasi and Tauseef, 2018) and Bhringaraj (Sawant et al., 2004), belonging to the genus *Eclipta* and family *Asteraceae* (Bánki et al., 2022).

### 3.2 Morphological characteristics and Paozhi (processing and preparation of botanical drugs)

**FIGURE 1:**
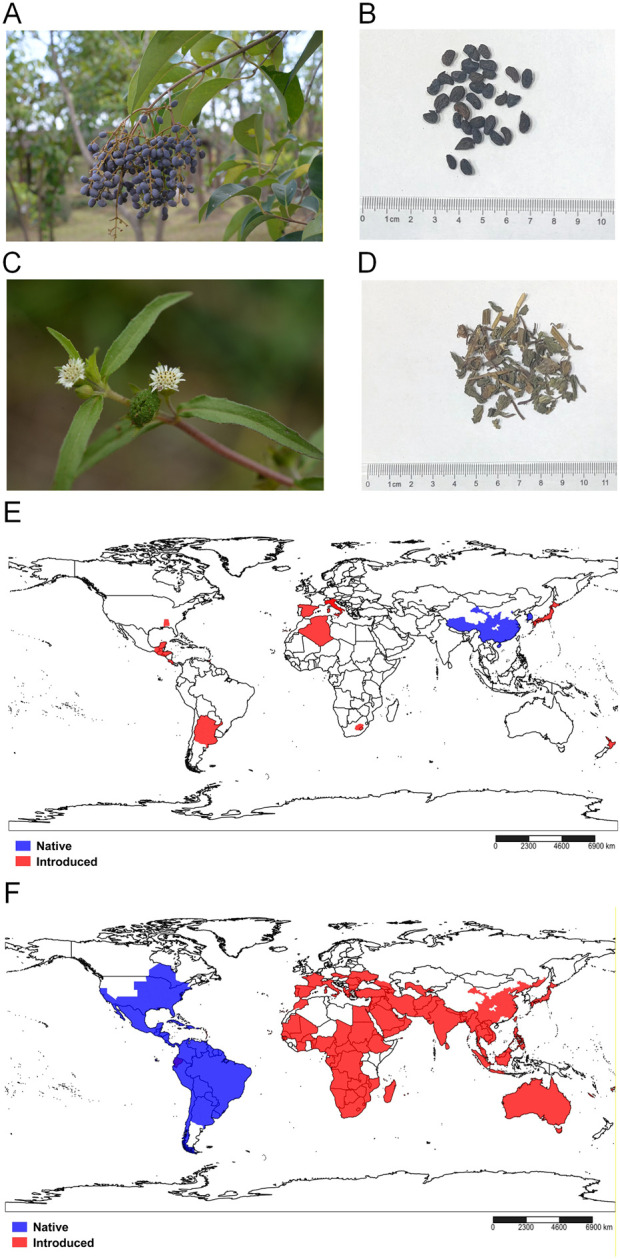
Comparison of the original plants, crude drugs, and global distribution of *Ligustrum lucidum* W.T.Aiton [Oleaceae; Ligustri Lucidi Fructus] (Hereafter referred to as Ligustri Lucidi Fructus) and *Eclipta prostrata* (L.) L. [*Asteraceae*; Ecliptae Herba] (Hereafter referred to as Ecliptae Herba). **(A)** Berries of Ligustri Lucidi Fructus in their natural state, highlighting their appearance and growth characteristics. **(B)** Harvested Ligustri Lucidi Fructus and its processed crude form prepared for medicinal use. **(C)** Ecliptae Herba source plant in its natural environment, illustrating its morphology. **(D)** Harvested and processed crude medicinal form of Ecliptae Herba, ready for medicinal use. **(E)** Global distribution of Ligustrum lucidum W.T.Aiton. (F) Global distribution of *Eclipta prostrata* (L.) L. Data sources included Species 2000 China, Catalogue of Life China, and Plants of the World Online (Kew Science). Data were translated into corresponding region codes using the SimpleMappr web tool, https://www.simplemappr.net/, to generate the image.

Harvested in winter when the fruit is ripe (Commission, 2025), Ligustri Lucidi Fructus is a deep blue–black fruit, ripening to a red–black hue, with a reniform or similar shape, 7–10 × 4–6 mm (Institute Of Botany, 1996). When dried, the fruit is 6–8.5 mm long and 3.5–5.5 mm in diameter, with a blackish–purple or grayish–black surface that is shrunken and uneven (Commission, 2025). Wine-steamed Ligustri Lucidi Fructus is used in Erzhi Pill. Clean Ligustri Lucidi Fructus is steamed with wine until the wine is entirely absorbed or evaporated (Commission, 2025). The berries and wine-steamed Ligustri Lucidi Fructus are shown in Figures 1A,B.

Collected at the flowering stage (Commission, 2025), *Eclipta prostrata* is a perennial (Tewtrakul et al., 2011) and aromatic (Lin et al., 2010) plant. The stems are erect or prostrate and heavily branched (Ananthi et al., 2003). The leaves are lanceolate, papery, and densely covered with rough hairs on both sides (Institute Of Botany, 1996). Small white daisy-like flowers (Abbasi and Tauseef, 2018) are organized in the capitulum approximately 6 mm wide (Institute Of Botany, 1996). The fruits are achenes, approximately 2.8 × 1.5 mm, with angular edges (Institute Of Botany, 1996). When dried, the intact leaves unfold into a long lanceolate shape that is shriveled and curled, with some leaves fragmented (Commission, 2025). Ecliptae Herba is then processed by eliminating the foreign matter, washing briefly, cutting into sections, and drying (Commission, 2025). The shapes of *Eclipta prostrata* and Ecliptae Herba are shown in Figures 1C,D.

### 3.3 Ecological habits

*Ligustrum lucidum* grows in predominantly warm-temperate climates (Dreyer et al., 2019) from 0 to 2900 m (Institute Of Botany, 1996) and is tolerant to air pollution, wind, poor soil (Montti et al., 2016), and shade (Wilcox, 2000). Ligustri Lucidi Fructus is natively produced in central and southern China (extending to Hainan) and southern Korea (Gardens, 2023); it is also distributed across all continents, except Antarctica (Dreyer et al., 2019). The geographic distribution of *Ligustrum lucidum*, including its native and introduced areas, is shown in Figure 1E.

*Eclipta prostrata* thrives in riversides, fields, abandoned ponds, and roadsides at altitudes less than 1,600 m (Institute Of Botany, 1996) and germinates over a wide range of soil temperatures (50°F–95°F) and pH values (5–8) (Prostko, 2012). Originally native to Central, North, and South America, it has been introduced into Africa, Asia, Australia, Europe, and the Pacific islands (Institute Of Botany, 1996). The worldwide distribution of *Eclipta prostrata* is shown in Figure 1F.

## 4 Comparison of the phytochemistry and quality control of Erzhi Pill and its constituent botanical drugs

### 4.1 Phytochemistry

As previously mentioned, the chemical profile and content of Ligustri Lucidi Fructus are modified through processing, which mitigates its cold-natured properties and enhances its therapeutic efficacy (Zhang et al., 2021; Luan et al., 2023). To establish a baseline for understanding these chemical transformations, we first systematically summarized the raw Ligustri Lucidi Fructus composition by identifying iridoids, triterpenoids, flavonoids, phenylethanols, and other metabolites (Supplementary Table S1). Specifically, we found that Ligustri Lucidi Fructus was characterized by a high abundance of iridoids, with secoiridoids representing the predominant subclass. Triterpenoids constituted a major class of bioactive metabolites in Ligustri Lucidi Fructus, comprising 5.61% of its total composition (Gao et al., 2015). In addition to iridoids, phenethyl alcohols also represented a distinctive and noteworthy class of metabolites in Ligustri Lucidi Fructus. Among these, iridoids, including specnuezhenide (nuezhenide) and nuezhenoside G13, and triterpenoids, including oleanolic acid and ursolic acid, were the main bioactive metabolites. Additionally, salidroside and hydroxytyrosol, as phenethyl alcohol metabolites, were shown to be very important active metabolites. In all, 188 metabolites have been identified in Ligustri Lucidi Fructus in previous studies. The structures of the principal active metabolites are provided in Supplementary Figure S1.

Phytochemical research on Ecliptae Herba has revealed the presence of triterpenoids, flavonoids, coumarins, thiophenes, polyacetylenes, and additional metabolites, with 134 metabolites currently documented (Supplementary Table S2). Notable bioactive metabolites include triterpenoids, such as ecliptasaponin A-B and eclalbasaponin I–II; flavonoids, such as apigenin and cosmosiin; coumarins, such as wedelolactone and demethylwedelolactone; steroids, including sitosterol and stigmasterol; and thiophenes, such as α-terthienyl. The structures of the main active metabolites in Ecliptae Herba are illustrated in Supplementary Figure S1B.

**FIGURE 2:**
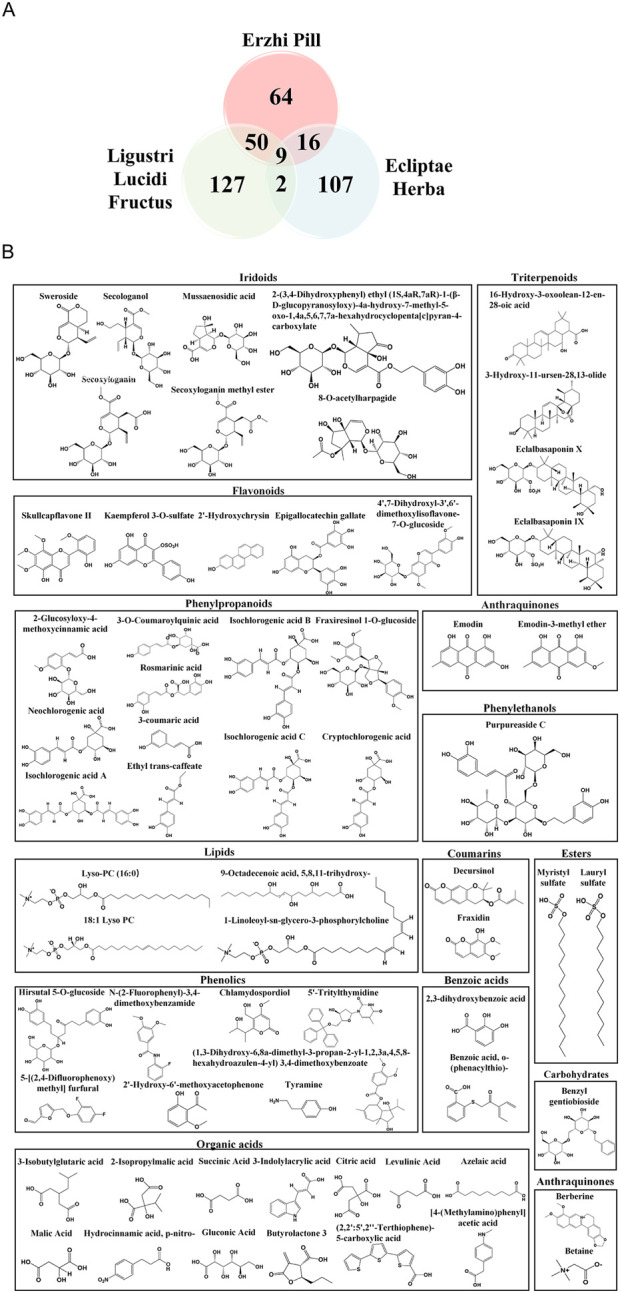
Comparison of metabolites and unique metabolite structural formulas in Ligustri Lucidi Fructus, Ecliptae Herba, and Erzhi Pill. **(A)** Comparison of metabolites in Ligustri Lucidi Fructus, Ecliptae Herba, and Erzhi Pill. **(B)** Structural formulas of 64 metabolites unique to Erzhi Pill compared with Ligustri Lucidi Fructus and Ecliptae Herba. Chemical structures were retrieved from the PubChem database and rendered using ChemDraw.

In Erzhi Pill, a total of 146 metabolites have been identified, including iridoids, triterpenoids, phenethyl alcohols, flavonoids, and coumarins (Supplementary Table S3), which represent the vast majority of the chemical metabolites of Ligustri Lucidi Fructus and Ecliptae Herba (excluding unidentified metabolites). To evaluate the distribution of metabolites among Ligustri Lucidi Fructus, Ecliptae Herba, and Erzhi Pill, a Venn diagram was constructed (Figure 2A). Notably, unique metabolites, including sweroside and secologanol, were identified in Erzhi Pill (Figure 2B). In contrast, 129 metabolites from Ligustri Lucidi Fructus and 109 from Ecliptae Herba were not identified in Erzhi Pill.

Although the included studies adopted reproducible methods, considerable discrepancies in the chemical profiles between Erzhi Pill and its constituent botanical drugs still exist. These differences can be attributed to three main factors. First, sample origin heterogeneity: the botanical sources of Ligustri Lucidi Fructus and Ecliptae Herba were not consistently authenticated or fully compliant with the *Chinese Pharmacopoeia* standards, leading to chemical variation caused by the geographical origin, plant variety, and processing methods. Second, analytical inconsistency: variations in extraction protocols, instrument precision, and data processing strategies resulted in divergent metabolite detection, especially for low-abundance metabolites, limiting cross-study comparability. Third, chemical transformation during formulation: the preparation of Erzhi Pill is not a simple mixture of single botanical drugs. Hydrolysis, complexation, and thermal degradation may occur, generating new metabolites unique to the formula and reducing the content of some original metabolites, which explains why many metabolites in single botanical drugs are absent in the final preparation.

To accurately characterize and compare the chemical metabolites of Erzhi Pill and its constituent botanical drugs, a standardized workflow is proposed. First, homogeneous authentic samples from Dao-di production areas should be used, authenticated by morphological and DNA barcoding methods, and prepared in line with the current *Chinese Pharmacopoeia* to ensure batch consistency. Then, high-resolution UPLC-Q-TOF-MS/MS in positive and negative ion modes should be applied to obtain comprehensive chromatographic fingerprints. Multivariate statistical analyses including principal component analysis (PCA) and Orthogonal Partial Least Squares Discriminant Analysis (OPLS-DA) can be used to screen differentially expressed, newly formed, or vanished metabolites. Finally, structural identification and validation should be carried out by comparing molecular weights, fragment ions, and retention times with reference standards, and simulated heating experiments using extracts of individual botanical drugs should be performed to verify the formation pathways of transformed metabolites during formulation.

### 4.2 Quality control and safety

Specnuezhenide had been established as the primary quality control marker for crude Ligustri Lucidi Fructus (Commission, 2025; Commission, 2020). However, the 2020 *Chinese Pharmacopoeia* revised the standard for the processed botanical drugs and instead designated salidroside as its quantitative marker (Commission, 2020; Commission, 2015; Commission, 2025). Research into the chemical metabolites of Ligustri Lucidi Fructus revealed that the content of specnuezhenide, its most abundant iridoid glycoside (Chu et al., 2018), was significantly reduced due to ester bond hydrolysis during wine steaming (Shang et al., 2021). In contrast, the content of salidroside, an absorbed metabolite with strong pharmacological activity, significantly increased (Li et al., 2020a; Shang et al., 2021; Zhang et al., 2021; Li et al., 2011b; Luan et al., 2023). These findings may be one of the reasons for the change in metabolite levels in the *Chinese Pharmacopoeia*. However, a single metabolite cannot accurately reflect the multi-active metabolite characteristics of Ligustri Lucidi Fructus. Recent studies simultaneously quantified 16 bioactive metabolites (Li et al., 2023), demonstrating a shift toward multiple metabolite quality assessments that better represent the complex nature of botanical drugs.

Similarly, wedelolactone serves as a characteristic marker for Ecliptae Herba, with the *Chinese Pharmacopoeia* specifying a minimum content of 0.040% (Commission, 2025). In addition to quantifying this single metabolite, a 2:1 ratio of wedelolactone to demethylwedelolactone has been proposed as a clinically relevant quality marker based on its nephroprotective effects (Wang et al., 2020), and a sensitive method for their simultaneous quantification in rat plasma has been developed (Wang et al., 2022). This signifies a conceptual advance in quality control by incorporating the interaction of biopotency and metabolites into quality assessment. Other researchers have proposed even more comprehensive multiple metabolite profiling systems in addition to ratios focused on limited markers. For example, Nguyen et al. suggested a set of 10 metabolites as chemical markers, including wedelolactone, luteolin, and apigenin, and established a corresponding quantification method (Nguyen et al., 2020). Han et al. used liquid chromatography–mass spectrometry to quantify nine characteristic metabolites in Ecliptae Herba samples from different regions, including luteolin and its glycosides and ecliptasaponin A (Han et al., 2015a). Sato et al. further proposed a bioactivity-guided standardization method based on sulfated flavonoids to increase the pharmacological relevance in quality control measures (Sato et al., 2024). These developments highlight the growing recognition that Ecliptae Herba efficacy results from synergistic interactions among multiple metabolites rather than isolated metabolites.

Multiple metabolite quantitative analysis has become important for evaluating the quality of TCM formulas. Relying solely on specnuezhenide as a quality marker for Erzhi Pill and Ligustri Lucidi Fructus is not sufficient. An antioxidant-oriented study on Erzhi Pill identified six key metabolites (salidroside, specnuezhenide, ligustroflavone, wedelolactone, oleanolic acid, and ursolic acid) that were used to generate an analytic hierarchy process (AHP)-based quality control model. The feasibility of the AHP model for the quality control of complex TCM systems and the suitability of these six monomers as chemical markers for Erzhi Pill were further validated through systematic *in vitro* experiments (Nie and Yao, 2018). In another study, by analyzing the fingerprints of Ligustri Lucidi Fructus (ethanol extract) and Ecliptae Herba (water decoction), eight characteristic markers were proposed for Erzhi Pill based on their abundance, prevalence, and pharmacological relevance. The markers included four metabolites of Ligustri Lucidi Fructus (specnuezhenide, 10-hydroxyoleoside dimethylester, salidroside, and verbascoside) and four metabolites of Ecliptae Herba (wedelolactone, ecliptasaponin A/D, eclalbasaponin C (or its isomer), and eclalbasaponin VI) (Jia et al., 2018). The above efficacy-guided multiple metabolite quality control strategy, which replaces the conventional method relying only on single-compound quantification, enables a more accurate evaluation of Erzhi Pill quality. Furthermore, the integration of multi-omics, network pharmacology, and other emerging approaches to establish a correlation between chemical composition, pharmacological activity, and clinical efficacy may further improve the precision of Erzhi Pill quality control. However, the wide application of such integrated strategies is still hindered by their methodological complexity and the lack of a unified consensus in the field. An overview of quantitative methods for Ligustri Lucidi Fructus, Ecliptae Herba, and Erzhi Pill is provided in Table 1.

**TABLE 1:** HPLC-based methods developed for the quantitative determination of Ligustri Lucidi Fructus, Ecliptae Herba, and Erzhi Pill.

Drugs	Components	Detection method	Standard	Ref.
Ligustri Lucidi Fructus	Specnuezhenide	HPLC	≥0.70%/g	Commission (2025)
	Salidroside	HPLC	≥0.20%/g	Commission (2025)
	Three Phenylethanoid glycosides three iridoids, seven flavonoids, and three triterpenoids	UHPLC-MS	Integrated score from PCA	Li et al. (2023)
Ecliptae Herba	Wedelolactone	HPLC	≥0.040%/g	Commission (2025)
	Wedelolactone (1) and demethylwedelolactone (2)	HPLC	Wedelolactone: demethylwedelolactone = 2:1	Wang et al. (2020)
	Chlorogenic acid (1), paratensein 7-O-beta-D-glucoside (2), quercetin 7-O-beta-D-glucoside (3), luteolin 7-O-beta-D-glucoside (4), apigenin 7-O-beta-D-glucoside (5), apigenin 4′-O-beta-D-glucoside (6), apigenin (7), luteolin (8), wedelolactone (9), and paratensein (10)	HPLC-PDA	–—	Nguyen et al. (2020)
	Luteolin 7-O-β-D-glucopyranoside (1), ecliptasaponin C (2), luteolin (3), eclalbasaponin IV (4), apigenin (5), ecliptasaponin A (6), echinocystic acid 28-O-β-D-glucopyranoside (7), echinocystic acid (8), and 3-oxo-16α-hydroxy-olean-12-en-28-oic acid (9)	LC-MS	–—	Han et al. (2015a)
	Seven sulfated flavonoids	LC-MS	–—	Sato et al. (2024)
Erzhi Pill	Specnuezhenide	HPLC	≥4.0 mg/g (Ligustri Lucidi Fructus)	Commission (2025)
	Salidroside (1), specnuezhenide (2), ligustroflavone (3), wedelolactone (4), oleanolic acid (5), and ursolic acid (6)	C_30_-HPLC	AHP	Nie and Yao (2018)
	Specnuezhenide (1), 10-hydroxyoleoside dimethylester (2), salidroside (3), verbascoside (4), wedelolactone (5), ecliptasaponin A/D (6), eclalbasaponin C (or its isomer) (7), and eclalbasaponin VI (8)	UHPLC/Q-Orbitrap-MS	–—	Jia et al. (2018)

HPLC, high-performance liquid chromatography; UHPLC-MS, ultra-HPLC-mass spectrometry; PCA, principal component analysis; HPLC-PDA, HPLC, equipped with photometric diode array detector; AHP, analytic hierarchy process.

Furthermore, the literature has indicated that Erzhi Pill does not exhibit acute toxicity in standardized tests. Specifically, in an acute oral toxicity study in which Erzhi Pill was administered at a dose of 60 g/kg, no acute toxicity was observed after 14 days of observation. This outcome confirmed the safety of Erzhi Pill in animal models at a dose of 60 g/kg, suggesting a favorable preliminary safety profile (Xie et al., 2021). However, systematic toxicity studies are still required to fully validate its safety for clinical use. These studies should include long-term toxicity evaluations to assess potential cumulative risks, reproductive toxicity studies for special populations, and *in vivo* investigations into metabolism-related hazards that may occur with prolonged administration.

## 5 Comparison of the efficacy and mechanisms of Erzhi Pill and constituent botanical drugs

**FIGURE 3:**
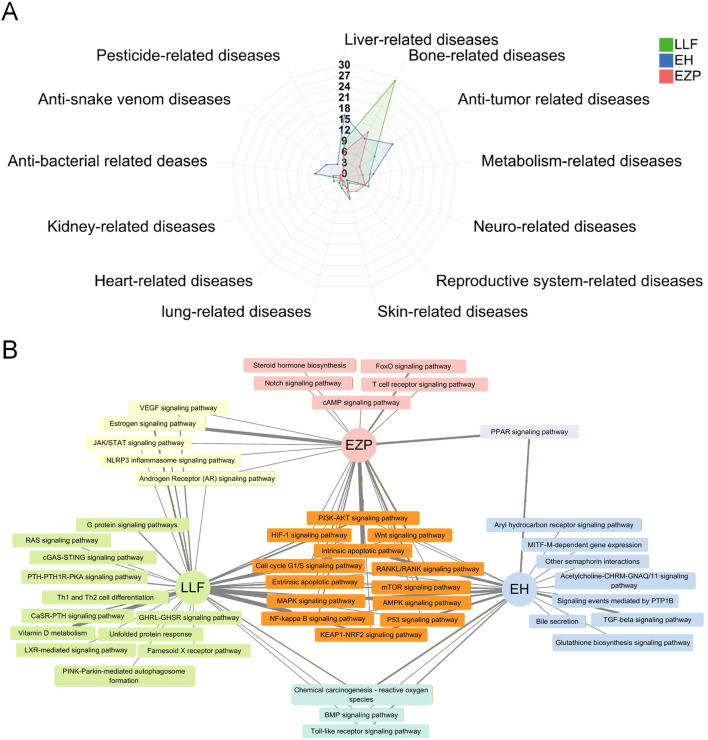
Comparative efficacy and mechanism of Ligustri Lucidi Fructus (LLF), Ecliptae Herba (EH), and Erzhi Pill (EZP). **(A)** Number of published articles. **(B)** Mechanistic pathways. Graphics were created using an online tool, https://www.chiplot.online/, and Cytoscape software.

The results of the literature analysis (Figure 3A) showed that Ligustri Lucidi Fructus exhibited efficacy in 11 disease categories, primarily skeletal disorders, and Ecliptae Herba (in studies limited to the above-ground part of the plant) alleviated symptoms across 13 types of diseases, mainly liver-related conditions. Erzhi Pill was reported to be effective in seven disease categories, with all pathologies entirely within the scope of those addressed by either botanical drug. These seven categories encompassed hepatic, skeletal, neoplastic, neurological, metabolic, reproductive, and dermatological diseases. The diseases and mechanisms related to Erzhi Pill treatment are listed in Table 2.

**TABLE 2:** Overview of pharmacological effects and mechanisms of Erzhi Pill.

Pharmacological activities	Experiment type	Models/Methods	Administration regimen	Effects and mechanisms	Erzhi Pill source	Ref.
Liver-related diseases	*In vivo* (male Wistar rats); negative control: normal control Positive control: rapamycin (1 mg/kg) for 14 days, starting at one-third partial hepatectomy	Experimental liver injury induced by 2-acetylaminofluorene (15 mg/kg intragastrically (i.g.) for 7 days), followed by standard one-third partial hepatectomy	1. Preventive group: Erzhi Pill (6.48 g/kg/d, i.g.), concurrent with 2-acetylaminofluorene exposure for 21 days 2. Therapeutic group: Erzhi Pill (6.48 g/kg/d, i.g), initiated after partial hepatectomy and continued for 14 days	Inhibiting the TSC/mTOR signaling pathway, reducing excessive hepatocyte apoptosis, and decreasing liver injury score	Batch no. Z32020882 from Lei Yun Shang Pharm, Shanghai, China	Zhou et al. (2017)
	*In vivo* (male Wistar rats); negative control: normal control Positive control: rapamycin (0.2 mg/kg) for 14 days, starting at one-third partial hepatectomy	Experimental liver injury induced by 2-acetylaminofluorene (15 mg/kg i.g. for 7 days), followed by standard one-third partial hepatectomy	1. Preventive group: Erzhi Pill (6.48 g/kg/d, i.g.), concurrent with 2-acetylaminofluorene exposure for 21 days 2. Therapeutic group: Erzhi Pill (6.48 g/kg/d, i.g.), initiated after partial hepatectomy and continued for 14 days	Inhibiting the PI3K-Akt-raptor-rictor pathway, downregulating the expression of apoptosis-related proteins, and reducing hepatocyte apoptosis	Batch no. Z32020882 from Lei Yun Shang Pharm, Shanghai, China	Zhao et al. (2018)
	*In vivo* (male C57BL/6J mice) Only negative control: normal control	1. Acute liver injury induced by 0.2% CCl_4_, 10 mL/kg, intraperitoneally (i.p.))	Erzhi Pill (0.6/1.2 g/kg/d, i.g.) pretreated for 4 weeks before modeling	Inhibiting Kupffer cell activation, reducing the release of inflammatory factors, and decreasing liver fibrosis degree	Neautus TCM, Sichuan, China: Ecliptae herba (Eclipta prostrata L., yerbadetajo herb) and glossy privet fruit (Ligustrum lucidum Ait., Ligustri lucidi fructus) by weight ratio (1:1), with Paeonia lactiflora Pall. (Radix Paeoniae Alba, debark peony root), Corbicula fluminea (Asian clam), and *Curcuma longa* L. (turmeric)	Zhao et al. (2019)
		2. Liver fibrosis induced by 10% CCl_4_ (1 mL/kg, i.p.) twice weekly for 8 weeks	Erzhi Pill (0.6/1.2 g/kg/d, i.g.) concurrent treatment from weeks 4–8			
		3. Liver cirrhosis induced by 10% 3,5-diethoxycarbonyl-1,4-dihydrocollidine (0.5 mg/kg, i.g.) twice weekly for 8 weeks	Erzhi Pill (0.6/1.2 g/kg/d, i.g.) concurrent treatment from weeks 4–8			
		4. Hepatocellular carcinoma induced by weekly diethylnitrosamine (165 mg/kg, i.p.) and CCl_4_ (1.5 mL/kg, i.g.) for 10 weeks (CCl_4_ was administered 3 days after each diethylnitrosamine dose.)	Erzhi Pill (0.6/1.2 g/kg/d, i.g.) pretreated for 4 weeks before modeling			
	*In vivo* (male Wistar rats) Only negative control: normal control	Acute liver injury induced by CCl_4_(1.5 mL/kg, i.p.) and assessed 2 h later	1. Preventive group: Erzhi Pill (18 g/kg/d, i.g.) for 7 days before modeling 2. Therapeutic group: Erzhi Pill (18 g/kg, i.g., twice within 12 h) starting 2 h after CCl_4_ injection for 7 days	Protecting the liver by regulating multiple metabolic pathways, including tryptophan and sphingolipid metabolism	A complex extract comprising a freeze-dried aqueous extract of *Herba Ecliptae* and the raw powder of *Fructus Ligustri Lucidi*	Yao et al. (2014)
Bone-related diseases	*In silico*	Network pharmacology	–—	Multitarget regulation of PI3K-Akt/MAPK pathways, inhibiting hepatocyte apoptosis and promoting liver tissue repair	–—	Huang et al. (2020b)
​	*In vivo* (female SD rats) Negative control: normal control Positive control: estradiol valerate (0.4 mg/kg/d, i.g.) starting on the 5th week after ovariectomy for 26 weeks	Osteoporosis model induced by bilateral ovariectomy	Erzhi Pill (9.0/4.5/2.25 g/kg/d, i.g.) from the 5th week after ovariectomy for 26 weeks	Preventing bone loss and improving bone quality by inhibiting osteoclastic bone resorption to restore bone metabolic balance	75% ethanol extract of Fructus Ligustri Lucidi, the fruit of *Ligustrum lucidum* Ait. (Oleaceae) and a water decoction of Herba Ecliptae, the aerial parts of *Eclipta prostrate* L. (Asteraceae), combined in equal volumes	Cheng et al. (2011)
	*In vivo* (female SD rats) Only negative control: normal control	Postmenopausal osteoporosis model induced by bilateral ovariectomy	Erzhi Pill (0.9 g/kg/d, i.g.) from 2 weeks after ovariectomy for 12 weeks	Improving bone mass and strength by activating the Sirt1/FoxO signaling axis and inhibiting PPARγ/microRNA-132 to balance bone metabolism	Ligustrum and Yerbadetajo Herb (1:1), sequentially extracted with 65% ethanol and water	Liang et al. (2018)
	*In vivo* (female C57BL/6 mice) Negative control: normal control Positive control: Estrogen (0.039 mg/kg/d, i.g.) for 8 weeks	*In vivo*: ovariectomy-induced perimenopausal osteoporosis	*In vivo*: Erzhi Pill (3.5/7 g/kg/d, i.g.) starting 1 week post-ovariectomy for 8 weeks	Improving estrogen levels and inhibiting bone loss by balancing the RANKL/OPG ratio and suppressing osteoclast differentiation	The arid aerial parts of E. prostrate L and the fruit of L. lucidum Ait. (Oleaceae) (1:1 by crude weight) with 70% and 80% ethanol	Qin et al. (2021)
	*In vitro* (bone marrow-derived monocytes from mice) Only negative control: normal control	*In vitro*: induced to differentiate into osteoclasts with 25 ng/mL macrophage colony-stimulating factor and 50 ng/mL RANKL	*In vitro*: wedelolactone, oleanolic acid, echinocystic acid, luteolin, and luteolin-7-O-glucoside at concentrations of 10^−7^–10^–5^ mol/L and Erzhi Pill with the medium changed every 3 days			
	*In vivo* (female SD rats) Only negative control: normal control	Alveolar bone osteoporosis model induced by bilateral ovariectomy	Erzhi Pill (1 g/kg/d, i.g.), from the 1 week after ovariectomy for 12 weeks	Protecting alveolar bone and regulating bone turnover by activating the Wnt/β-catenin signaling pathway	Aqueous extract of a 1:1 mixture of *Herba Ecliptae* and *Fructus Ligustri Lucidi*	Sun et al. (2014)
	*In vivo* (male SD rats) Negative control: normal control Positive control Methotrexate (1.5 mg/kg i.g. twice a week), 28 consecutive days	*In vivo*: 50 µL of emulsified bovine type II collagen with isopycnic incomplete Freund’s adjuvant was intradermally injected at the tail root	*In vivo*, Erzhi Pill monotherapy: 1.8 g/kg i.g., twice daily, 28 consecutive days	Alleviating rheumatoid arthritis and protecting bone by activating the Wnt/β-catenin pathway and synergizing with methotrexate to enhance osteoblast function	Yaodu Zhangshu Pharmaceutical Co., Ltd, Jiangxi, China	Li et al. (2020b)
​	*In vitro* (UMR-106 cells) Negative control: normal control medicated serum Positive control: methotrexate medicated serum	*In vitro*: treated with serum from collagen-induced arthritis rats and drug-administered rats	*In vitro* serum treatment: UMR-106 cells cultured with medium containing 15% medicated serum for 48 h	​	​	​
	*In vivo* (male SD rats) Only negative control: normal control	*In vivo*: administered Erzhi Pill to prepare the drug-containing serum	Erzhi Pill (3.6 g/kg, i.g.) twice daily for 7 days	Salidroside, an active component of Erzhi Pill, promoting the proliferation and differentiation of ROB	The wine-steamed fruit of Ligustrum lucidum Ait. and aqueous extract of the aerial parts of Eclipta prostrate L	Gao et al. (2022)
	*In vitro* (ROB cells from SD rats) Only negative control: normal control	*In vitro*: ROB cells	Salidroside: 0.1/1/10/100/1,000 nM for 48 h			
	*In vivo* (male SD rats) Only negative control: normal control	*In vivo*: administered Erzhi Pill to prepare the drug-containing serum	*In vivo*: Erzhi Pill (0.45/1.8/7.2 g/kg/d, i.g.) twice daily for seven total doses	Specifically targeting osteoclasts by suppressing their proliferation and differentiation, consistent with kidney yin nourishment	A 1:1 (w/w) aqueous extract of processed *Fructus Ligustri Lucidi* and *Herba Ecliptae*	Zhang et al. (2008a)
	*In vitro* (primary osteoblasts from the rats/UMR106 cells/RAW264.7 cells) Only negative control: normal control	*In vitro*: RAW264.7 cells induced to differentiate into osteoclasts with 25 ng/mL RANKL and 30 ng/mL macrophage colony-stimulating factor for 6 days	*In vitro*: drug-containing serum added to the cell culture medium, incubated for 24–72 h (proliferation) or 4–6 days (osteoclast differentiation)			
	*In vitro* (primary osteoblasts from SD rats) Only negative control: normal control	*In vitro*: primary osteoblasts	Ethanolic extract/volatile compounds from Erzhi Pill (1–100 μg/mL), incubated for 48 h (proliferation) or 9 days (alkaline phosphatase)	Promoting osteoblast proliferation and differentiation via active components (triterpene acids and volatile compounds)	75% ethanol-reflux extract/volatile oil of the aerial parts of *Eclipta prostrasta* L. and the fruits of *Ligustrum lucidum* Ait	Wu et al. (2012)
	*In vitro* (bone marrow mesenchymal stem cells from female BALB/c mice) Only negative control: normal control	*In vitro*: bone marrow mesenchymal stem cells	*In vitro*: wedelolactone (3/6/9 μM) combined with oleonuezhenide (4.5/9/18 μM), incubated for 2 days (proliferation), 9 days (alkaline phosphatase), and 21 days (mineralization)	Synergistically promoting bone formation and alleviating cytotoxicity by co-activating both canonical and non-canonical Wnt signaling pathways	—	Deng et al. (2019)
	*In vivo* (female C57BL/6 mice) Only negative control: normal control	*In vivo*: ovariectomy osteoporosis model	*In vivo*: wedelolactone 10 mg/kg and oleonuezhenide 50 mg/kg, i.p., every 2 days for 1 month (initiated 2 days post-ovariectomy)			
​	*In silico*	Network pharmacology, molecular docking, and molecular dynamics	—	Alleviating osteoporosis through multicomponent synergy (e.g., flavonoids and triterpenoids) targeting core proteins (e.g., ER1 and TRAF6) to regulate osteoclast differentiation and hormone-related pathways	—	Li et al. (2022)
	*In silico* *In vivo* (AB strain zebrafish (wild-type and transgenic sp7: enhanced green fluorescent protein)) Only negative control: normal control	*In silico*: network pharmacology *In vivo*:10 μM dexamethasone-induced bone loss for zebrafish	Quercetin treatment: zebrafish larvae were exposed to 0.5–16 μM quercetin for 6 days	Erzhi Pill mediates anti-osteoporotic effects by multitarget pathway regulation (PI3K-Akt, TNF, and interleukin-17) and quercetin specifically reverses glucocorticoid-induced osteoblast suppression	—	Zhong et al. (2021)
	*In silico* *In vivo* (AB strain zebrafish) Negative control: normal control; positive control: etidronate disodium (15 μM) for 7 days	*In silico*: network pharmacology and molecular docking *In vivo*: 25 μM prednisolone-induced glucocorticoid-induced osteoporosis in zebrafish	Zebrafish larvae exposed to Erzhi Pill (0.1/1.0/10.0 μg/mL) for 7 days	Alleviating glucocorticoid-induced osteoporosis via multicomponent, multitarget, multi-pathway mechanisms and improving bone mineralization and locomotion in zebrafish	50% ethanol reflux extract of a 1:1 (w/w) mixture of *Herba Ecliptae* and *Fructus Ligustri Lucidi*	Cai et al. (2022)
Antitumor related diseases	*In silico*	*In silico*: network pharmacology	—	Multicomponent and multitarget co-regulating PI3K-Akt/TNF-α/p53 pathways to exert antibreast cancer activity	—	Duan et al. (2021)
	*In silico*	*In silico:* network pharmacology and molecular docking	—	Combating hepatocellular carcinoma via multicomponent synergy (e.g., luteolin, quercetin) by targeting senescence and cell cycle pathways to inhibit proliferation and predict prognosis	—	Zheng and Pan (2022)
​	*In vivo* (female SD rats) Only negative control: normal control	*In vivo*: administered Erzhi Pill to prepare the drug-containing serum	*In vivo*: Erzhi Pill (5.406 g/kg, i.g.) twice daily for 3 consecutive days	Suppressing triple-negative breast cancer progression by inhibiting proliferation, migration, and EMT through PPARγ pathway activation	Batch no. Z36020854 from Jiangxi Renfeng Pharmaceutical Co., Ltd., Jiangxi, China	Qi et al. (2025)
	*In vitro* (triple-negative breast cancer cells: MDA-MB-231/468 cells) Only negative control: normal control	Rescue experiment: 10 μM 2-chloro-5-nitrobenzanilide pretreatment for 2 h	*In vitro* treatment: cultured with 0%/5%/10%/20% Erzhi Pill-containing serum for 24–72 h; *In vitro* rescue experiment: co-cultured with 20% Erzhi Pill-containing serum for 48 h			
	*In vitro* (A375 cells/B16F10 cells /Jurkat cells) Only negative control: normal control	*In vitro*: 10 ng/mL interferon-γ-induced PD-L1-high A375 cells; co-culture with Jurkat cells	*In vitro*: Erzhi Pill extract 0.001–1 μg/mL, 24–72 h	Downregulating JAK-STAT/NF-κB pathways, inhibiting PD-L1, inducing melanoma apoptosis, and enhancing T-cell cytotoxicity	Batch no. Z36021716 from Jiangxi Yaodu Zhangshu Pharmaceutical Co., Ltd, Jiangxi, China	Fang et al. (2024)
	*In vivo* (female C57BL/6 mice) Negative control: normal control Positive control Dacarbazine (70 mg/kg, i.p.) every 2 days from day 4 until day 14 In silico	*In vivo*: Subcutaneous injection of 6 × 10^4^ B16F10 cells in mice *In silico*: network pharmacology	*In vivo*: Erzhi Pill (0.59/1.17/2.34 g/kg/d, i.g.) for 14 consecutive days			
Metabolism-related diseases	*In silico* *In vivo* (male SD rats) Only negative control: normal control	*In silico*: network pharmacology *In vivo*: diabetic cardiomyopathy model induced by 4-week high-fat diet feeding and streptozotocin (35 mg/kg in 0.1 mol/L citrate buffer, pH 4.5, i.p.)	Erzhi Pill (1/2/4 g/kg/d, i.g.) for 8 weeks	Ameliorating diabetic cardiomyopathy by modulating cardiac metabolism, attenuating oxidative stress and inflammation, and inhibiting cardiomyocyte apoptosis, mainly via regulating AMPK and PI3K/Akt/FoxO pathways	Reflux extraction of a 1:1 mixture of Ligustri Lucidi Fructus and Ecliptae Herba with 70% ethanol	Peng et al. (2022)
	*In vivo* (female SD rats) Negative control: normal control Positive control Irbesartan (15 mg/kg/d, i.g.) for 16 weeks	Diabetic nephropathy model induced by 4-week high-sugar/high-fat diet and streptozotocin (40 mg/kg in citrate buffer, pH 4.5, i.p.)	Erzhi Pill (15/10/5 g/kg/d, i.g.) for 16 weeks	Alleviating renal injury and proteinuria in diabetic nephropathic rats by upregulating podocyte CD2AP/podocin and suppressing inflammation and oxidative stress, with mild glucose-lowering effects	80% aqueous ethanol reflux extract of Ecliptae Herbs and Ligustri Lucidi Fructus	Jiang et al. (2018)
​	*In vivo* (male C57BL/6 mice) Negative control: normal control Positive control Resmetirom (15 mg/kg) same schedule as Erzhi Pill	*In vivo*: Non-obese metabolic dysfunction-associated steatohepatitis model: methionine-choline-deficient diet for 8 weeks; obese metabolic dysfunction-associated steatohepatitis mode: high-fat high-cholesterol diet for 24 weeks	*In vivo*: Erzhi Pill (1.5/3/6 g/kg/d, i.g) last 4 weeks of an 8-week methionine-choline-deficient diet or last 12 weeks of a 24-week high-fat high-cholesterol diet Salidroside (80 mg/kg) and echinocystic acid-3-o-glucoside (20 mg/kg, 4:1 ratio, i.g.): last 12 weeks of a 24-week high-fat high-cholesterol diet	Coordinately improving hepatic steatosis, insulin resistance, inflammation, apoptosis, and fibrosis through multitarget modulation of PPARα, PI3K/AKT, NLRP3, p53, and YAP pathways	Batch no. 230104 from Jiangxi Yaodu Zhangshu Pharmaceutical Co., Ltd., Zhangshu, China	Gao et al. (2025b)
	*In vitro* (HepG2/LX-2/BMDMs from the mice/THP-1); only negative control: normal control *In silico*	*In vitro* HepG2 cells: steatosis induced by 1.2 mM free fatty acid (oleic acid: palmitic acid = 2:1) for 24 h LX-2 cells: fibrosis induced by 10 ng/mL TGF-β1 for 24 h; BMDMs/THP-1 cells: NLRP3 activation induced by lipopolysaccharide-primed and ATP/nigericin for 4 h *In silico*: molecular docking	*In vitro*: wedelolactone (40 μM), specnuezhenide (80 μM), salidroside (80 μM), echinocystic acid-3-o-glucoside (20 μM), and the combination of salidroside (80 μM) with echinocystic acid-3-o-glucoside (20 μM, 4:1 ratio)			
	*In silico*	*In silico*: network pharmacology	—	Multicomponent regulation of PI3K-Akt/MAPK pathways to ameliorate lipid accumulation and insulin resistance	​	Huang et al. (2020a)
Neuro-related diseases	*In vivo* (male SD rats) Negative control: normal control Positive control: vitamins E and C (0.06 g/kg/d, i.g.) for 6 weeks	Aging model induced by thymectomy (1 mL/100 g intraperitoneal injection) and D-galactose (200 mg/kg/day IP) for 6 weeks	Erzhi Pill (4.32 g/kg/d, i.g.) for 6 weeks	Alleviating aging-related oxidative damage and neuronal apoptosis by enhancing mitochondrial membrane potential and systemic antioxidant capacity	Ligustri lucidi and Herba Ecliptae processed into a solution, by extraction and concentration	Gao et al. (2010)
	*In vivo* (female SD rats) Negative control: normal control Positive control: oestradiol valerate (0.80 mg/kg/d, i.g.) from day 22 to day 56 (for 35 days)	Alzheimer’s disease model was established by bilateral ovariectomy on day 0, followed by D-galactose (100 mg/kg/d, i.p.) starting on day 8, and Aβ1-40 (10 μg/rat, i.p.) on day 36	Erzhi Pill (1.5/0.75 g/kg/d, i.g.) from day 22 to day 56 (for 35 days)	Combats Alzheimer’s disease by restoring estrogen, clearing *Aβ*/p-tau, and activating the PI3K/Akt pathway to enhance neuronal survival	Jiangxi Yaodu Zhangshu Pharmaceutical Co., Ltd. (Jiangxi, China)	Xie et al. (2021)
​	*In vivo* (male C57BL/6 mice) Negative control: normal control Positive control Estradiol Valerate (0.468 g/kg/d, i.g.), same schedule as the Erzhi Pill	Cognitive impairment induced by lipopolysaccharide (0.25 mg/kg/d i.p.) for 1 week	1. Erzhi Pill (2.43 g/kg/d, i.g.) for 2 weeks (1-week pretreatment and 1-week co-treatment) 2. Apigenin and wedelolactone: 20 mg/kg each, same schedule as the Erzhi Pill	Primarily through apigenin and wedelolactone, activates the ERβ/TFAP2A pathway to enhance tyrosine hydroxylase-mediated norepinephrine synthesis, thereby alleviating cognitive impairment	A 1:1 mixture of *Ligustrum lucidum* and *Eclipta prostrata*) prepared by decocting 200 g of each herb (1:8, w/v)	Xue et al. (2025)
	*In silico*	*In silico*: network pharmacology, molecular docking and molecular dynamics	—	Alleviating Alzheimer’s pathology via blood–brain barrier-permeable components (e.g., apigenin) that multitarget Aβ/tau clearance and neuroinflammation through PI3K-Akt/p53 pathways	—	Yu et al. (2024)
	*In vitro* (primary cortical neurons from Wistar rats) Only negative control: normal control *In silico*	*In vitro*: apoptosis and synaptic damage induced by dexamethasone (10^–6^ mol/L) incubated for 72 h *In silico*: network pharmacology	Pretreatment with Erzhi Pill (0.1–10 μg/mL), followed by co-incubation with dexamethasone for 72 h	Exerting antidepressant-related neuroprotective effects by inhibiting neuronal apoptosis, restoring synaptic structure, and function, and modulating the 11β-HSD1-glucocorticoid/glucocorticoid receptor signaling pathway	A 1:1 mixture of the 70% ethanol extracts from Ligustrum lucidum Ait (Oleaceae Hoffmanns; Ligustri lucidi fructus) and Eclipta prostrata L (Asteraceae Bercht; Ecliptae herba)	Han et al. (2023c)
	*In silico*	*In silico*: reverse network pharmacology, molecular docking, and molecular dynamics	—	Demonstrating multitarget neuroprotective potential against depression via core components (quercetin and luteolin) targeting SPP1/SERPINE1 and modulating p53 signaling	—	Lu et al. (2023)
Reproductive system-related diseases	*In vitro* (BPH-1 cells) Only negative control: normal control	*In vitro*: 100 nM 17β-estradiol-induced cell proliferation	*In vitro*: ligustroflavonic acid/echinocystic acid (10 nM/100 nM/1 μM)	Suppressing BPH via multitarget regulation of the AR/ER/SRD5A pathway and inhibition of PCNA, mediated by Erzhi Pill and its active components (ligustrosidic acid and echinocystic acid)	Ethanol extract (80% and 70%) of *Ligustri Lucidi Fructus and Ecliptae Herba* (1:1)	Tao et al. (2022)
	*In vivo* (male Wistar rats) Negative control: normal control Positive control: flutamide (30 mg/kg/d, i.g.) for 45 days	*In vivo*: 21 days post-castration, rats received subcutaneous 17β-estradiol/testosterone (1:100 M ratio, corn oil, 10 μg/1,000 g) for 45 consecutive days to induce BPH	*In vivo*: Erzhi Pill (1.5/3/6 g/kg/d, i.g.) for 45 days			
​	*In vivo* (female C57 mice) Negative control: normal control Positive control: dehydroepiandrosterone (6 mg/kg/d, i.g.) same schedule as the Erzhi Pill	*In vivo*: premature ovarian failure model induced by a single intraperitoneal injection of cyclophosphamide (100 mg/kg)	*In vivo*: Erzhi Pill (2 g/kg/d, i.g.) for 28 days (7-day pretreatment and 21-day post-modeling)	Alleviating premature ovarian failure by activating ESR1 to enhance follicular development and inhibit granulosa cell apoptosis, primarily through specnuezhenide and ecliptasaponin A	Dissolving *Ligustrum lucidum Ait* and *Eclipta prostrata* powders in distilled water	Xu et al. (2025)
	*In vitro* (KGN cells) Only negative control: normal control *In silico*	*In vitro*: 250 μM cyclophosphamide for 48 h to induce premature ovarian failure *In silico*: network pharmacology	*In vitro*: cyclophosphamide and specnuezhenide (5 μg/mL)/ecliptasaponin A (25 μM)/combination			
	*In silico*	*In silico*: network pharmacology	—	Alleviating menopausal syndrome via targeting estrogen signaling, HIF-1, and PI3K-Akt axes to regulate hormonal balance and oxidative stress	—	Xu et al. (2012)
	*In vivo* (female ApoE-deficient C57BL/6 mice) Negative control: normal control Positive control: atorvastatin (3.03 mg/kg, i.g.) for 12 weeks	Postmenopausal atherosclerosis model: after ovariectomy, immediate Western high-fat diet feeding post-operation, 12 weeks	Erzhi Pill (0.7/1.4 g/kg/d, i.g.) for 12 weeks	Alleviating postmenopausal atherosclerosis by regulating estrogen levels to improve lipid metabolism and balance antioxidant capacity	—	Dai et al. (2018)
Skin-related diseases	*In vitro* (immortalized human keratinocytes) Only negative control: normal control	*In vitro*: ultraviolet radiation b (60 mJ/cm^2^)-induced photoaging	*In vitro*: Erzhi Pill 10^–3^/10^–2^/10^–1^ mg/mL) pretreatment 24 h	Alleviating photoaging by activating the Nrf2/HO-1/NQO1 pathway to suppress oxidative stress and reduce apoptosis	80% ethanol extract of Erzhi Pill (Beijing Tongrentang Co., Ltd., Beijing China)	Liu et al. (2022)
	*In vivo* (male ICR mice) Only negative control: normal control	*In vivo*: dorsal depilation (2 × 3 cm^2^), ultraviolet radiation A (94.52 J/cm^2^) and ultraviolet radiation B (9.45 J/cm^2^) 5 times/week for 9 weeks	*In vivo*: Erzhi Pill (0.67/1.33 g/kg), on the depilated dorsal skin before irradiation for 9 weeks			
	*In vivo* (Zebrafish) Negative control: normal control Positive control forskolin (5 μM) for 24 h	*In vivo*: Zebrafish pretreated with 0.2 mM propylthiouracil for 24 h to induce depigmentation	*In vivo*: Zebrafish embryos exposed to Erzhi Pill (0.03/0.1/0.5/1 mg/mL) for 24 h	Promoting melanin synthesis by activating the cAMP/PKA-CREB signaling pathway to upregulate MITF and tyrosinase expression	An aqueous extract was prepared from a 1:1 mixture of *L. lucidum* W.T. Aiton *and E. prostrata (L.)* L	Hong et al. (2024)
​	*In vivo* (C57BL/6 mice) *In vitro* (B16F10 cells) Negative control: normal control Positive control forskolin (5 μM) for 48 h	*In vivo*: C57BL/6 mice administered Erzhi Pill to prepare the drug-containing serum *In vitro*: B16F10 melanogenesis model	*In vivo*: C57BL/6 mice received Erzhi Pill (4 g/kg twice daily) for 3 days *In vitro*: 5%/10%/15% Erzhi Pill-containing serum, 48 h	​	​	​
	*In vivo* (female C57BL/6 mice) Negative control: normal control Positive control cyclosporin A (12.5 mg/kg, subcutaneously (s.c.)) for the final 4 weeks	*In vivo*: dorsal depilation (2 × 2 cm^2^), 40% monobenzone cream (16 weeks) and 18-h restraint stress (every 3 days)	*In vivo*: Erzhi Pill (2.57 g/kg, i.g.) for once every other day for the final 4 weeks	Alleviating chemical-induced depigmentation by inhibiting the MIF-CD74-NF-κB pathway to suppress oxidative stress and CD8^+^ T-cell-mediated inflammation	Containing *Ligustri Lucidi Fructus* extract and *Ecliptae Herba*	Tang et al. (2024)
	*In vitro* (immortalized human keratinocytes) Negative control: normal control Positive control ISO-1 (10 μM) for 24 h *In silico*	*In vitro*: 400 μM monobenzone and 10 ng/mL MIF for 24 h *In silico*: network pharmacology	*In vitro*: specnuezhenide (12.5/25/50 μM) for 24 h			
	*In vitro* (ARPE-19/HUVEC cells) Only negative control: normal control *In silico*	*In vitro:* ARPE-19 cells treated with 200 μM H_2_O_2_ for 12/24 h to establish an AMD-associated oxidative stress model; HUVEC cells cultured with conditioned medium from H_2_O_2_-treated ARPE-19 cells *In silico*: network pharmacology	*In vitro:* 5%/10%/15% Erzhi Pill-containing serum pretreatment	Alleviating age-related macular degeneration by inhibiting the HIF-1α/STAT3 pathway to suppress oxidative stress, inflammation, and pathological angiogenesis	—	Ding et al. (2025)
Other pharmacological properties	*In vivo* (female SD rats) Only negative control: normal control	*In vivo*: administered Erzhi Pill to prepare drug-containing serum	*In vivo*: Erzhi Pill (3.6 g/kg, i.g.) twice daily for 7 days	Alleviating cellular senescence by restoring amino acid and lipid metabolism homeostasis, primarily through regulating glutamine/glutamate metabolism and phospholipid biosynthesis pathways	A 1:1 mixture of wine-steamed *Ligustrum lucidum* Ait. fruit powder and lyophilized aerial parts of *Eclipta prostrata* L. extract	Shang et al. (2024)
	*In vitro* (BRL cells/NRK cells) Only negative control: normal control	*In vitro*: Transwell co-culture: upper (BRL cells, 20% blank serum, 48 h preculture); lower (NRK cells, 20 mg/mL D-galactose, 48-h preculture)	*In vitro*: transwell co-culture: upper (BRL cells, 20% Erzhi Pill-containing serum, 48 h preculture); lower (NRK cells, 20 mg/mL D-galactose, 48-h preculture)			

i.g., intragastrically; i.p., intraperitoneally; s.c., subcutaneously; TSC, tuberous sclerosis complex; mTOR, mammalian target of rapamycin; PI3K, phosphoinositide 3-kinase; Akt, protein kinase B; raptor, Regulatory-Associated Protein of mTOR, Complex 1; Rictor, Rapamycin-Insensitive Companion of mTOR; CCl_4_, carbon tetrachloride; MAPK, mitogen-activated protein kinase; SD, standard deviation; Sirt1, sirtuin 1; FoxO, Forkhead Box O; PPAR, peroxisome proliferator-activated receptor; RANKL, Receptor Activator of Nuclear Factor-κB ligand; OPG, osteoprotegerin; TRAF6, TNF, Receptor-Associated Factor 6; TNF, tumor necrosis factor; P53, Tumor Protein P53; EMT, epithelial–mesenchymal transition; PD-L1, Programmed Death-Ligand 1; JAK, janus kinase; STAT, signal transducer and activator of transcription; AMPK, adenosine monophosphate-activated protein kinase; CD2AP, CD2-associated protein; NLRP3, NOD-Like Receptor Pyrin Domain-Containing Protein 3; YAP, Yes-Associated Protein; Aβ, amyloid beta; ER, estrogen receptor; TFAP2A, Transcription Factor Activation Protein-2 Alpha; 11β-HSD1, 11β-Hydroxysteroid Dehydrogenase Type 1; SPP1, Secreted Phosphoprotein 1; SERPINE1, Serpin Family E Member 1; AR, androgen receptor; SRD5A, Steroid 5α-Reductase; HIF, hypoxia-inducible factor; Nrf2, Nuclear Factor Erythroid 2-Related Factor 2; HO-1, heme oxygenase-1; NQO1, NAD (P) H:Quinone Oxidoreductase 1; cAMP/PKA, cyclic adenosine monophosphate/protein kinase A; CREB, cAMP-Response Element-Binding Protein; PCNA: proliferating cell nuclear antigen; MITF, microphthalmia-associated transcription factor; MIF, macrophage migration inhibitory factor; NF-κB, Nuclear Factor-Kappa B.

The plant materials used to prepare Erzhi Pill in the included studies were verified. Despite minor variations in description across the literature, all references were confirmed to denote the same species using the Kew Medicinal Plant Names Service (MPNS). The materials are as follows: Ligustri Lucidi Fructus, the fruit of *Ligustrum lucidum* W.T.Aiton, from the genus *Ligustrum* and the family *Oleaceae*; and Ecliptae Herba, the aerial part of *Eclipta prostrata* (L.) L., from the genus *Eclipta* and the family *Asteraceae*.

### 5.1 Liver-related diseases

Erzhi Pill and its constituent botanical drugs Ligustri Lucidi Fructus and Ecliptae Herba have been reported to exert protective effects against acute liver injury and hepatic fibrosis. Ligustri Lucidi Fructus mitigated the adverse effects of acute liver injury induced by carbon tetrachloride (CCl_4_) (Hu et al., 2022) and arachidonic acid-iron (Seo et al., 2017) and attenuated hepatic fibrosis (Qin et al., 2024). Ecliptae Herba was shown to be effective against acute liver injury induced by CCl_4_ (Singh et al., 2001; Lu et al., 2016) and tetrachloroethylene (Singh et al., 1993). Multiple active monomers, including wedelolactone (Xia et al., 2013), luteolin (Jeong et al., 2013), luteoloside (Xiu et al., 2023), chinocystic acid, and eclalbasaponin II (Lee et al., 2008), were shown to exert protective effects against liver fibrosis through diverse mechanisms. In studies evaluating the protective effects of Erzhi Pill against CCl_4_-induced liver injury, we focused primarily on identifying potential biomarkers, with the evaluation of the efficacy limited to demonstrating improvements in pathological liver damage (Yao et al., 2014). Moreover, the administered dose of 18 g/kg/d was far higher than the human equivalent dose, which compromises the reliability and credibility of the results (Yao et al., 2014). A different study reported no significant effect on alanine aminotransferase (ALT) or aspartate aminotransferase (AST) levels (Zhao et al., 2019). The results in fibrosis models have not been promising: Erzhi Pill failed to improve ALT and AST levels or *in situ* tumor necrosis factor (TNF)-α and transforming growth factor (TGF)-β levels in the liver (Zhao et al., 2019). Although this study implemented detailed quality control measures, the presence of other medicinal metabolites in the formula, such as Paeonia lactiflora Pall., implies that the reported results cannot accurately reflect the efficacy of Erzhi Pill in treating liver injury.

Therefore, research on the hepatoprotective effects of Erzhi Pill remains insufficient. Future research should, under quality-controlled conditions, utilize multiple models of liver injury to compare the pharmacodynamic differences between the Erzhi Pill formula and its individual constituent botanical drugs (at twice the dose) in order to determine whether its compatibility confers a hepatoprotective advantage.

Erzhi Pill and Ligustri Lucidi Fructus (Zhang et al., 2025) were both shown to ameliorate drug-induced liver injury (DILI). Two experimental studies from the same research team demonstrated that Erzhi Pill ameliorated DILI induced by 2-acetylaminofluorene and partial hepatectomy through tuberous sclerosis complex/mechanistic target of rapamycin (TSC/mTOR) signaling and the phosphatidylinositol 3-kinase/protein kinase B/regulatory-associated protein of mTOR/rapamycin-insensitive companion of mTOR (PI3K/Akt/raptor/rictor) pathway (Zhou et al., 2017; Zhao et al., 2018). Virtual screening by another group also predicted the potential efficacy of Erzhi Pill against DILI, with the PI3K/Akt pathway identified as one of the core pathways (Huang et al., 2020b). While encouraging efficacy results have been observed at four times the human equivalent dose, the single-dose design employed in this study limits the determination of its minimum effective dose. Subsequent studies employing graded dose administration will help more precisely establish its efficacy threshold. In addition, virtual screening by another group also predicted its potential efficacy against DILI, and the PI3K/Akt pathway was one of the core pathways (Huang et al., 2020b).

Additionally, although Erzhi Pill has been shown to exhibit anti-cirrhotic activity, the effect was not significant (Zhao et al., 2019). Cholestatic liver injury (Wang et al., 2024a) and immune-mediated hepatitis (Luo et al., 2018) are unique pharmacological effects of wedelolactone, a quality control marker of Ecliptae Herba.

Overall, research on the efficacy of Ligustri Lucidi Fructus in acute liver injury is relatively extensive. Ecliptae Herba has also demonstrated significant activity in acute liver injury and liver fibrosis. Evidence from high-quality basic research on the efficacy of Erzhi Pill in improving liver-related diseases remains relatively limited, and DILI may represent a promising therapeutic condition, such as mitigating methotrexate-induced liver injury.

### 5.2 Bone-related diseases

Research on these three agents has primarily focused on osteoporosis; this condition has shown the most significant results and remains the most deeply investigated among the diseases treated by Erzhi Pill.

Ligustri Lucidi Fructus has demonstrated significant ameliorative effects in rat models of hormonal osteoporosis (ovariectomy-induced) (Zhang et al., 2008b), senile osteoporosis (Deng et al., 2025), disease-induced osteoporosis (diabetes-induced (Feng et al., 2019)), and oxidative stress-related osteoporosis (Wu et al., 2021) and in a zebrafish model of ferric ammonium citrate-induced osteoporosis (Jiang et al., 2024). Ligustri Lucidi Fructus also increased bone mineral density and improved bone properties in normal growing rats (Feng et al., 2014; Lyu et al., 2014). The underlying mechanism primarily involved calcium homeostasis regulation. Numerous studies further confirmed that extracts and active monomers of Ligustri Lucidi Fructus regulated the differentiation and function of osteoblasts (Wu et al., 2021; Qin et al., 2023; Li et al., 2015; Li et al., 2010; Kong et al., 2022; Ko et al., 2010) and osteoclasts (Xu et al., 2016; Ko et al., 2010). The mechanisms were centered on modulating calcium homeostasis and involved classical pathways, including the osteoprotegerin/receptor activator of nuclear factor-κB ligand (OPG/RANKL) pathway. One study uniquely demonstrated that Ligustri Lucidi Fructus improved inflammation in rats with lumbar intervertebral disk protrusion, alleviating pain (Han et al., 2015b).

Research on the use of Ecliptae Herba in bone-related diseases is less extensive and less in-depth than on Ligustri Lucidi Fructus. Only two articles reported its use in maintaining bone homeostasis in ovariectomized rats (Zhang et al., 2013b; Deng et al., 2015). Studies indicated that the extracts (Lin et al., 2010) and active monomers (Lee et al., 2009) of Ecliptae Herba, particularly wedelolactone (Deng et al., 2018), enhanced osteoblast differentiation and function while suppressing osteoclastogenesis via several pathways, including the Wnt/β-catenin (Liu et al., 2016) and nuclear factor κ-B (NF-κB)/c-fos/nuclear factor of activated T cells 1 pathways (Liu et al., 2016).

Erzhi Pill ameliorated bone loss and improved bone properties in both ovariectomy-induced (Cheng et al., 2011; Liang et al., 2018; Qin et al., 2021; Sun et al., 2014) and glucocorticoid-induced (Zhong et al., 2021; Cai et al., 2022) osteoporosis animal models through mechanisms involving the sirtuin 1/forkhead box O (Sirt1/FoxO) axis, Wnt/β-catenin, and PI3K signaling, and by modulating the RANKL/OPG ratio. Erzhi Pill also regulated osteoblast and osteoclast activities to counteract bone loss in primary cultures and cell lines (Gao et al., 2022; Zhang et al., 2008a; Wu et al., 2012). Interestingly, one study evaluated the rationale of the traditional composition of Erzhi Pill by combining wedelolactone and oleonuezhenide, which showed enhanced anti-osteoporotic effects in an ovariectomized mouse model and osteoblasts by upregulating Wnt5a signaling and reducing cytotoxicity in bone mesenchymal stem cells (Deng et al., 2019). Furthermore, our team expanded its application by demonstrating that Erzhi Pill potentiated methotrexate to ameliorate bone loss in collagen-induced arthritis rats through osteoblastic Wnt/β-catenin activation, without observable inhibitory effects on osteoclasts (Li et al., 2020b).

Since there are currently no specific first-line drugs for the treatment of osteoporosis, most of the included studies did not set up positive controls, with only a few using estrogen as a positive control. Although the dosages varied across the included studies, they all fell within a reasonable range. Therefore, the evidence supporting the anti-osteoporotic activity of Erzhi Pill is reliable. An important question worth investigating is in which type of osteoporosis does Erzhi Pill have a therapeutic advantage in treating so as to enable development of precision medicine.

### 5.3 Anti-tumor related diseases

Although Ligustri Lucidi Fructus exhibits inhibitory effects on various tumors, most studies have been focused on the cellular level. Only one study reported its suppressive effect on tumor growth in a glioma mouse model (Jeong et al., 2011). Ligustri Lucidi Fructus has been shown to inhibit proliferation in multiple cancer cell types, including liver cancer (Hu et al., 2014; Yan et al., 2010), colon cancer (Zhang et al., 2011; Li et al., 2002), leukemia (Zhang et al., 2007), fibrosarcoma (Kim et al., 2022), glioma (Jeong et al., 2011), lung cancer cells (Wang et al., 2014), and in tumors expressing ATP-binding cassette subfamily C member 1 (Braga et al., 2007). The mechanisms of Ligustri Lucidi Fructus primarily involved cell cycle arrest and the activation of multiple cell death pathways. Ligustri Lucidi Fructus also showed potential for alleviating chemotherapy-induced bone marrow suppression (Han et al., 2023a). However, because cellular experiments are removed from the complex *in vivo* microenvironment and lack neuro-immune-endocrine regulation and metabolic processes, the results should only be considered suggestive.

Ecliptae Herba and its active metabolites also exhibited inhibitory activity against various tumors. Both *in vivo* and *in vitro* studies demonstrated the significant anticancer efficacy of Ecliptae Herba and its active metabolites against liver cancer (Chaudhary et al., 2011; Liu et al., 2012; Chaudhary et al., 2013; Chaudhary et al., 2014), breast cancer (Arya et al., 2015; Li et al., 2024a), and multiple myeloma (Li et al., 2024b). Ecliptae Herba also mitigated tumor-induced hepatorenal toxicity and drug resistance in liver cancer (Arya et al., 2015). *In vivo* evidence supported its preventive and therapeutic effects on skin cancer through tumor protein 53 (p53)-mediated apoptosis (Ali et al., 2014). *In vitro* studies further confirmed the antiproliferative and pro-apoptotic actions of Ecliptae Herba against conditions such as ovarian cancer (Cho et al., 2016; Kim et al., 2015; Preya et al., 2017), glioma (Chaudhary et al., 2011), renal carcinoma (Chaudhary et al., 2011), head and neck squamous cell carcinoma (Zou et al., 2022), and chemotherapy-induced alopecia (Wang et al., 2024b). Mechanistically, Ecliptae Herba inhibited proliferation through cell cycle arrest, promoted apoptosis via pathways such as the p53 and Kelch-like ECH-associated protein 1/nuclear factor erythroid 2-related factor 2 (Nrf2)/heme oxygenase-1 (HO-1) pathway, modulated cell migration through TGF-β1/Smad family member signaling, and promoted oxidative stress-induced cytotoxicity.

Studies on the antitumor effects of Erzhi Pill are limited. One animal study demonstrated its ability to inhibit melanoma tumor growth through NF-κB and T-cell receptor signaling pathways (Fang et al., 2024). However, this study only employed cellular experiments for evaluation and lacked a positive control group. To enhance the credibility of the findings, it would be advisable to conduct multidose *in vivo* experiments that include a positive control group. Both *in vivo* and *in vitro* experiments showed that Erzhi Pill exhibited efficacy comparable to that of cisplatin in triple-negative breast cancer by activating the peroxisome proliferator-activated receptor (PPAR) γ pathway via carboxylesterase 1 (Qi et al., 2025). Network pharmacology analyses further suggested that Erzhi Pill may exert therapeutic effects on breast cancer (Duan et al., 2021) and liver cancer (Zheng and Pan, 2022) by modulating pathways such as PI3K/Akt and by regulating senescence-related genes.

The anticancer activity of Erzhi Pill has been primarily attributed to Ecliptae Herba. Based on the TCM theory that Erzhi Pill nourishes the liver and kidneys (kidneys govern reproduction in TCM), as well as current research, liver cancer and breast cancer are potential indications. However, given that Ligustri Lucidi Fructus alleviates bone marrow suppression and Ecliptae Herba mitigated drug resistance and chemotherapy-induced alopecia, Erzhi Pill may be more accurately positioned as an adjuvant therapy in cancer treatment.

### 5.4 Metabolism-related diseases

Erzhi Pill, Ligustri Lucidi Fructus, and Ecliptae Herba have all demonstrated pharmacological efficacy against glycolipid metabolism disorder-related diseases, particularly non-alcoholic fatty liver disease (NAFLD), lipid-induced vascular diseases, and diabetes. In NAFLD, Ligustri Lucidi Fructus reduced triglyceride accumulation in hepatic tissue and cells by inhibiting lipid synthesis factors (Yang et al., 2015; Zhang et al., 2013a), while Ecliptae Herba enhanced liver function and improved lipid profiles in rat models (Hussein et al., 2018; Helmy et al., 2019). A similar efficacy for Erzhi Pill was predicted by applying virtual screening methods (Huang et al., 2020b) and verified through rigorous and reliable animal and cell models on metabolic dysfunction-associated steatohepatitis, which is more severe than NAFLD (Gao et al., 2025b).

Furthermore, all three formulations are reported to exert antidiabetic effects, albeit through distinct mechanisms. For example, while both Ligustri Lucidi Fructus and Ecliptae Herba were shown to regulate blood glucose levels, Ligustri Lucidi Fructus acted by targeting insulin resistance and protecting islet β-cells (Yang et al., 2020), whereas Ecliptae Herba modulated glycolysis and gluconeogenesis (Ananthi et al., 2003). Additionally, Ligustri Lucidi Fructus ameliorated diabetic nephropathy and diabetic retinopathy through several mechanisms, including the restoration of glucose and lipid levels (Luan et al., 2023), inhibition of the cyclic GMP-AMP synthase (cGAS)-stimulator of interferon gene pathway (Liu et al., 2025), and suppression of the hypoxia inducible factor (HIF)-1α/vascular endothelial growth factor signaling pathway (Wu et al., 2016a). Erzhi Pill shows mild glucose-lowering effects, but available research suggests that it exerted protective effects against diabetic cardiomyopathy and diabetic nephropathy through the AMP-activated protein kinase (AMPK) and PI3K/Akt/FoxO3a signaling pathways (Peng et al., 2022) and by modulating podocin and CD2-associated protein (CD2AP) protein levels and mRNA expression (Jiang et al., 2018). However, the study on diabetic cardiomyopathy lacks a positive control, and the highest dose used in the diabetic nephropathy study was almost 10 times the human equivalent dose. These findings still require support from more rigorously designed experiments.

In lipid-induced vascular diseases, Ligustri Lucidi Fructus alleviated lipid-oxidative stress injury of blood vessels through c-Jun N-terminal kinase and mitogen-activated protein kinase (MAPK) signaling pathways (Gao et al., 2025a), whereas Ecliptae Herba exerted anti-atherosclerotic effects by promoting vasodilation (Prachayasittikul. et al., 2010). In addition, Ecliptae Herba demonstrated efficacy in models of obesity and hyperlipidemia (Dhandapani, 2007; Le et al., 2021; Yao et al., 2022). There are no research reports on the use of Erzhi Pill in regulating lipid-induced vascular diseases.

Overall, in basic research on the improvement induced by Erzhi Pill in metabolic diseases, only one evidence regarding its effect on metabolic dysfunction-associated steatohepatitis is highly reliable (Gao et al., 2025b). This evidence not only confirms its efficacy at multiple doses but also delves into the exploration of active metabolites and their mechanisms. Evidence in studies on other glucose and lipid metabolism diseases is relatively scarce, and positive results should be interpreted with caution. For credible investigations into the effects of Erzhi Pill on metabolic diseases, the approach of Gao et al. is highly worthy of reference (Gao et al., 2025b).

### 5.5 Neurological-related diseases

Animal studies of Parkinson’s disease models demonstrated that Ligustri Lucidi Fructus has neuroprotective efficacy comparable to that of selegiline (Cai et al., 2012). Ligustri Lucidi Fructus active metabolites restored impaired synaptic plasticity in a transgenic Alzheimer’s mouse model (Luo et al., 2020), ameliorated neuroinflammation associated with depression-like behaviors (Feng et al., 2020), and regulated neuroendocrine factor secretion in aged rats (Cai et al., 2011). *In vitro* evidence indicated that Ligustri Lucidi Fructus promoted neuronal function and prevented apoptosis by inducing extracellular signal-regulated kinase and cAMP response element-binding protein (CREB) phosphorylation and inhibiting the nitric oxide pathway (Fu et al., 2010; Li et al., 2011a).

In contrast to Ligustri Lucidi Fructus, Ecliptae Herba has exhibited a distinct spectrum of neuropharmacological activity in studies, with demonstrated efficacy in ameliorating ischemic stroke (Tao et al., 2025; Mansoorali et al., 2012), exerting antiepileptic effects (Tambe et al., 2017; Muke et al., 2018; Shaikh et al., 2013), and mitigating cholinergic blockade-induced cognitive impairment (Jung et al., 2018). Virtual screening and network pharmacology further suggested that Ligustri Lucidi Fructus has therapeutic potential in Alzheimer’s disease (Wei et al., 2023). Mechanistic studies have indicated that its neuroprotective effects are primarily mediated by inhibiting oxidative damage and modulating pathways such as HIF-1α/solute carrier family 7 member 11/glutathione peroxidase 4 and Akt/glycogen synthase kinase-3-β signaling.

Research on use of Erzhi Pill for treatment of neurological disorders is limited and has predominantly focused on Alzheimer’s disease. Integrated evidence from network pharmacology, proteomics, and animal studies across multiple research groups has consistently identified the critical role of the PI3K/Akt signaling pathway in mediating the ability of Erzhi Pill to improve learning and memory abilities in Alzheimer models (Xie et al., 2021; Yu et al., 2024). One research group demonstrated that Erzhi Pill restored estrogen receptor-β (ERβ), transcription factor AP-2α (TFAP2A), and tyrosine hydroxylase transcriptional activity in the context of Alzheimer’s pathology (Xue et al., 2025). Although this study adopted a preparation method different from the Erzhi Pill specified in the *Chinese Pharmacopoeia* and evaluated its efficacy using only a single dose, the results can still, to some extent, support the therapeutic potential of Erzhi Pill for Alzheimer’s disease.

Erzhi Pill was also shown to inhibit cerebral cortex neuronal apoptosis in both rat senescence and cortical neuron injury models (Gao et al., 2010; Han et al., 2023c). By applying reverse network pharmacology, Erzhi Pill was further predicted to treat depression of the Yin deficiency type (Lu et al., 2023). The efficacy of Erzhi Pill in Alzheimer’s disease has been experimentally supported or computationally predicted across these studies. Through the multitarget mechanisms of its constituent botanical drugs, Erzhi Pill may provide a unique therapeutic or preventive intervention for Alzheimer’s disease.

### 5.6 Reproductive system-related diseases

Erzhi Pill and its constituent botanical drugs have been reported to exert ameliorative effects on benign prostatic hyperplasia (BPH), premature ovarian failure, and menopausal syndrome through mechanisms primarily associated with hormone-like activity. Ligustri Lucidi Fructus and Ecliptae Herba found in Erzhi Pill ameliorate BPH (Tao et al., 2022) and premature ovarian failure (Xu et al., 2025) in animal models, primarily through mechanisms associated with the estradiol pathway. *In vitro* studies also confirmed that multiple active metabolites in Ligustri Lucidi Fructus regulated ovarian granulosa cells by influencing estradiol secretion (Cai and Luo, 2024), while extracts of Erzhi Pill activated the estrogen response element reporter system without stimulating MCF-7 cell proliferation (Xu et al., 2012). In addition, in an ovariectomized apolipoprotein E-deficient mouse model, Erzhi Pill increased estrogen levels and the uterine index, with positive effects against postmenopausal cardiovascular pathologies (Dai et al., 2018).

Partly due to challenges in replicating animal and cellular models of reproductive diseases and difficulties in evaluating model validity and pharmacological efficacy, only limited research is available on the therapeutic effects of Erzhi Pill on reproductive system-related diseases aligned with the TCM theory that “the kidney is the foundation of innate constitution” and its property of nourishing kidney Yin. Erzhi Pill efficacy in treating menopausal syndrome has also been demonstrated in clinical practice. Unlike its multitarget actions in other diseases, both Erzhi Pill and its constituent botanical drugs consistently regulate estrogen-related indicators in reproductive system-related diseases. Identifying the chemical basis for the estrogen-modulating effects of Erzhi Pill and determining the optimal ratio of active metabolites may maximize its efficacy in treating menopausal syndrome and other reproductive disorders.

### 5.7 Skin-related diseases

Erzhi Pill and its constituent botanical drugs have demonstrated efficacy against vitiligo through distinct mechanisms. Active monomers of Ligustri Lucidi Fructus and extracts of Ecliptae Herba were reported to promote melanogenesis and melanocyte migration by enhancing intracellular actin cytoskeleton aggregation (Wu et al., 2016b) and microphthalmia-associated transcription factor expression (Xu et al., 2017). Erzhi Pill elevated the melanin content of melanoma cells, zebrafish, and mouse models by activating the cyclic adenosine monophosphate/protein kinase A (cAMP/PKA) signaling pathway (Hong et al., 2024), thus suppressing DNA oxidative damage and cell death (Tang et al., 2024). Additionally, Ecliptae Herba extracts and the ethanol extract of Erzhi Pill protected against UV-induced skin damage and senescence *in vitro* by inhibiting oxidative stress (Chan et al., 2014; Liu et al., 2022). Network pharmacology and cellular experiments suggest that Erzhi Pill may ameliorate age-related macular degeneration by suppressing oxidative stress, inflammation, and angiogenesis in the retinal pigment epithelium (Ding et al., 2025). Ligustri Lucidi Fructus exhibited skin-whitening effects through anti-inflammatory activity (Guo et al., 2021), while the efficacy of Ecliptae Herba was comparable to that of minoxidil in hair loss treatment (Roy et al., 2008; Begum et al., 2014; Begum et al., 2015).

The experimental evidence for the efficacy of Erzhi Pill in improving vitiligo symptoms is credible, but autoimmune dysregulation is central to the pathogenesis of vitiligo, and current evidence does not indicate that Erzhi Pill modulates melanocyte-specific autoimmune responses. If future studies confirm that Erzhi Pill has potent immunoregulatory properties, it may be a promising therapeutic candidate for vitiligo.

### 5.8 Other pharmacological properties

The above sections cover the main therapeutic areas in the fundamental research on Erzhi Pill. However, additional studies revealed that Ligustri Lucidi Fructus and Ecliptae Herba have also demonstrated antioxidant effects (Ju et al., 2012; Han et al., 2014; Wu et al., 2020), alleviated pulmonary fibrosis (Wu et al., 2024; Yang et al., 2019; You et al., 2015), exerted antibacterial (Wu et al., 2015; Tran et al., 2024; Shrawani and Verma, 2022) and antiviral effects (Ma et al., 2001; Tewtrakul et al., 2007), and promoted wound healing (Xi et al., 2024; Linh et al., 2024). Specifically, Ligustri Lucidi Fructus demonstrated anti-aging effects (Wang et al., 2025), mitigated nephrotoxicity (Chen et al., 2021), counteracted renal fibrosis (Zhang et al., 2023), and modulated immune responses (Wang et al., 2012; Liu et al., 2021). Ecliptae Herba provided unique protection against myocardial injury (Ge et al., 2024) and exhibited anti-snake venom activity (Melo et al., 1994; Melo and Ownby, 1999; Pithayanukul et al., 2004) and mosquito-repellent properties (Govindarajan and Sivakumar, 2012; Govindarajan and Sivakumar, 2011; Govindarajan and Karuppannan, 2011; Govindarajan, 2011).

Current studies indicated that Erzhi Pill has pharmacological activity against multiple diseases, primarily due to its multiple metabolites and multitarget characteristics. With its traditional effects of nourishing the liver and kidneys and based on the TCM theory of “kidney governing essence and bones,” Erzhi Pill theoretically may provide advantages as a therapeutic agent for liver diseases, reproductive system disorders, and bone-related conditions. Basic research studies have confirmed its potential efficacy in DILI, demonstrated effectiveness against osteoporosis, and shown mechanistic consistency in treating reproductive system diseases.

Oxidative stress is a fundamental pathological mechanism underlying various tissue damages and aging-related processes. Numerous natural and synthetic metabolites have demonstrated tissue-specific antioxidant properties. For instance, the ethanolic extract of propolis exhibits significant hepatoprotective effects against oxidative stress in liver tissue (Selamoglu et al., 2015), while synthetic organoselenium compounds show potent antioxidative activity in both the lung and kidney tissues (Talas et al., 2009). Additionally, natural flavones such as Jaceosidin have been recognized for their versatile pharmacological activities including antioxidant stress potentials (Nageen et al., 2021). Fungal species also contribute to this field; studies on *Lepista nuda* have demonstrated its antioxidant capacity (Bal et al., 2019), and *Fomitopsis pinicola* has been investigated for its therapeutic potential in beneficial dietary approaches (Sevindik et al., 2017). These findings highlight the importance of exploring tissue-specific antioxidant effects from diverse sources.

Although Erzhi Pill has not been specifically reported to mitigate oxidative damage in tissue, multiple pharmacological studies have examined its inhibition of oxidative stress processes. However, whether its antioxidant effects exhibit tissue specificity and how its activity compares with those of the aforementioned agents remain to be further evaluated. Combined with its mild properties and the ability to regulate estrogen levels (Figure 3B), Erzhi Pill may have potential in anti-aging application in women by mitigating antioxidant damage (Shang et al., 2024).

Based on its protective efficacy against DILI, positive effects on bone homeostasis, and regulation of glucose and lipid metabolism, Erzhi Pill could be hypothetically combined with methotrexate to reduce methotrexate-induced hepatotoxicity, enhance bone protection, and alleviate metabolic disorders associated with rheumatoid arthritis.

However, several issues remain in Erzhi Pill research. Although numerous studies have focused on the efficacy of single active metabolites, more research similar to that by Sun et al. (2023) is needed to identify the active metabolites in Ligustri Lucidi Fructus and Ecliptae Herba that act synergistically. These investigations may clarify the material basis of Erzhi Pill and validate the rationality of combination treatments. After clarifying its material basis, the ratio of multiple active metabolites can be optimized to further enhance the efficacy of Erzhi Pill and overcome problems caused by the inherently unstable nature of raw botanical drug quality in the formulation. Additionally, while the literature has revealed that the water extract of Ecliptae Herba induced acute toxicity in mice when administered at doses exceeding 2 g/kg/day (Singh et al., 2013), Erzhi Pill, in contrast, was confirmed to cause no acute toxicity. This conclusion was drawn from standardized acute toxicity tests conducted at a dose of 60 g/kg/day (Xie et al., 2021). However, the underlying reasons for this discrepancy in toxicity between the two remain unclear. It is essential to determine the reasons for this difference to further understand the safety of single-botanical-drug extracts compared with their corresponding metabolite preparations and to provide a theoretical basis for the rational clinical application of Erzhi Pill.

## 6 Clinical applications

### 6.1 Comparison of traditional applications

The earliest record of Ligustri Lucidi Fructus as a treatment is in the *Shen Nong Ben Cao Jing*, an ancient manuscript dating back over 1,000 years, which describes Ligustri Lucidi Fructus as follows: “It supplements the middle, pacifies the five organs, nourishes the spirit, and eliminates various illnesses. Long-term use promotes physical fitness and delays aging.” The efficacy of Ligustri Lucidi Fructus mainly derives from its ability to nourish Yin by entering the kidneys and liver. By enhancing kidney and liver function, Ligustri Lucidi Fructus improves Xu Fa Zao Bai (premature graying of the beard and hair); Mu An Bu Ming (blurred vision); Xuan Yun Er Ming, Yao Xi Suan Ruan, and Nei Re Xiao Ke (internal heat and consumptive thirst); and Gu Zheng Chao Re (steaming bone tidal fever) in TCM theory (Commission, 2025). The *Korean Pharmacopoeia* does not separately list Ligustri Lucidi Fructus; instead, influenced by the TCM theory, it is categorized as a “Yin-tonifying botanical drug” and primarily used for patterns of Liver–Kidney Yin deficiency.

*The Tang Materia Medica* (659 CE) first documented Ecliptae Herba under the name “Li Chang” and explicitly described its hemostatic properties as “effective against bloody dysentery,” which emphasized its hemostatic activity. Ecliptae Herba, similar to Ligustri Lucidi Fructus, primarily exerts its therapeutic effects by tonifying the liver and kidneys. This botanical drug is clinically indicated for Liver-Kidney Yin deficiency syndromes, including Xu Fa Zao Bai, Xuan Yun Er Ming, and Yao Xi Suan Ruan. Distinct from Ligustri Lucidi Fructus, the sour and cold properties of Ecliptae Herba provide additional blood-cooling and hemostatic activities. Ecliptae Herba has demonstrated broad efficacy in managing hemorrhagic disorders caused by Yin deficiency with blood-heat, including bleeding due to oral, nasal, urethral, anal, or vaginal hemorrhage and traumatic bleeding (Commission, 2025). In Brazil, Ecliptae Herba is a traditional medicine known as erva-botâo and is widely used to treat pulmonary disorders, diarrhea, syphilis, snakebites, filariasis, and leprosy (Morel et al., 2024; Morel et al., 2017; Rajakumar and Abdul Rahuman, 2011; Khanna et al., 2009; Mors et al., 1989). In the *Ayurvedic Pharmacopoeia of India*, the whole plant is specified for managing hepatic diseases and hyperlipidemia (Kumari et al., 2006). It is also widely used in other countries, with ethnomedicinal uses in Bangladesh, India, Nepal, and Pakistan reported by Jahan et al. (2014), which are not discussed here.

The name Erzhi Pill is derived from the two solar terms, the summer solstice (Xia Zhi) and winter solstice (Dong Zhi), which correspond to the optimal harvest times for Ecliptae Herba and Ligustri Lucidi Fructus, respectively. This temporal symbolism reflects a deep philosophical alignment with the natural balance of Yin and Yang in TCM. The combination of Ligustri Lucidi Fructus and Ecliptae Herba in Erzhi Pill represents a deliberate application of TCM formulation principles and is designed to achieve synergistic enhancement in a classical example of medicinal “partnering.” Individually, Ligustri Lucidi Fructus with a cool and bitter nature strongly nourishes Liver–Kidney Yin, while Ecliptae Herba, with cold and sour properties, tonifies the liver and kidneys, cools blood, and stops bleeding. In combination, the actions of each integrate and potentiate one another: the nourishing effect of Ligustri Lucidi Fructus is stabilized and extended by the astringency of Ecliptae Herba (sourness governs astringency), while the cooling and hemostatic functions of Ecliptae Herba are grounded and enhanced by the cool nature of Ligustri Lucidi Fructus. The resulting formulation preserves the individual strengths of each botanical drug and treats a broader range of symptoms with greater efficacy. Specifically, the synergy of the metabolites in Erzhi Pill enhances its ability to nourish Yin, clear deficiency heat, and reinforce the liver and kidney more effectively than administering either herb alone. Moreover, Erzhi Pill introduces new therapeutic applications, such as significantly alleviating dryness of the throat and nose (Yan Gan Bi Zao), which are minimally resolved by each metabolite alone. This exemplifies the “1 + 1 > 2” effect central to sophisticated TCM metabolomics, rendering Erzhi Pill particularly suitable for patterns of Liver–Kidney Yin deficiency with heat signs or mild bleeding tendencies. Grounded in the TCM principles of “mutual reinforcement of Yin and Yang” and “liver-kidney homology,” Erzhi Pill embodies the philosophy of leveraging medicinal synergy to achieve holistic regulation. A comparative summary of the ethnopharmacological uses of Ligustri Lucidi Fructus, Ecliptae Herba, and Erzhi Pill is provided in Table 3.

**TABLE 3:** Comparison of the ethnopharmacological effects of Ligustri Lucidi Fructus, Ecliptae Herba, and Erzhi Pill (Commission, 2020).

Medicinal profile	Ligustri lucidi fructus	Ecliptae herba	Erzhi Pill
Property and flavor	Sweet		^a^
	Cool and bitter	Cold and sour	
Meridian tropism	Liver meridian and kidney meridian		
Actions	Tonifying the liver and kidney		
	Improving vision blackening hairs	Blood-cooling hemostatic action	Nourishing yin stopping bleeding
Indications	Liver-kidney yin deficiency; Xuan Yun Er Ming (vertigo/dizziness and tinnitus) Yao Xi Suan Ruan (soreness and weakness of the lower back and knees)		
	Xu Fa Zao Bai (premature graying of beard/hair)		—
	Mu An Bu Ming (blurred vision); Nei Re Xiao Ke (internal heat and consumptive thirst); Gu Zheng Chaore (steaming bone tidal fever)	Ya Chi Song Dong (loose teeth); Tu Xue (hematemesis), Bi Niu (epis-taxis), Niao Xue (hematuria), Xue Li (bloody dysentery), and Beng Lou Xia Xue (menstrual flooding) caused by yin deficiency and blood heat; traumatic bleeding	Yan Gan Bi Zao (dryness in the throat and nose) Yue Jing Liang Duo (heavy menstruation)

^a^ In the TCM, system, the prescription has no property and flavor and meridian tropism.

### 6.2 Modern applications

No clinical studies have evaluated Ligustri Lucidi Fructus or Ecliptae Herba as a single botanical drug. Both are exclusively used in metabolite prescriptions containing multiple botanical drugs. Consequently, their clinical therapeutic efficacy cannot be compared with that of Erzhi Pill. Similarly, due to the predominant focus of TCM practitioners on clinical practice, there was little retrievable English literature on Erzhi Pill and its constituent botanical drugs relative to their extensive clinical use. Furthermore, as many diseases often present with complex patterns beyond Liver–Kidney Yin deficiency, Erzhi Pill is commonly used as a foundation to develop derivative formulas in clinical practice.

Menopausal syndrome is caused by the decline in ovarian function and fluctuations in sex hormone levels and is characterized primarily by menstrual irregularities and vasomotor dysfunction. TCM attributes the fundamental etiology of menopausal symptoms to kidney deficiency, with kidney Yin deficiency pattern predominating clinically (Huazhang and Chunyan, 2008). Core symptoms include Gu Zheng Chaore, Xuan Yun Er Ming, Yao Xi Suan Ruan, and menstrual disorder. As expected, studies on TCM usage patterns revealed that the combination of the botanical drugs “*Ligustrum lucidum* with *Eclipta prostrata*” was among the most frequently used botanical drug pairs for treating menopausal syndrome (Yang et al., 2009; Chen et al., 2011). More compelling evidence showed that Erzhi Pill combined with other formulas and acupuncture achieved an efficacy rate of 96.83% for menopausal syndrome with kidney Yin and Yin–Yang deficiency patterns, significantly higher than the 73.33% effective rate observed with Fuchun capsules alone (a Western medication) (Huazhang and Chunyan, 2008). A rigorous randomized, double-blind, placebo-controlled study found that compared with the baseline and the control group, a modified Erzhi Pill treatment group demonstrated significant improvements in all five parameters evaluating vaginal atrophy (Chen et al., 2018). In addition another randomized, double-blind, placebo-controlled trial found that Erzhi Pill with Dan Zhi Qing Re formula modulates serum estrogen levels and vasomotor symptoms, respectively (Fu et al., 2016). These findings were consistent with the established understanding in modern medicine that menopausal syndrome involves hormonal fluctuations and vasomotor dysfunction. Furthermore, this result was consistent with the fundamental research findings related to the involvement of Erzhi Pill on the estrogen signaling pathway. While there is also the highest level of evidence supporting the use of Erzhi Pill for the treatment of menopausal syndrome (Chen et al., 2018; Fu et al., 2016), the limitation is that all direct evidence involves formulations modified from Erzhi Pill or its combination with other medications or therapeutic approaches. Therefore, the role of Erzhi Pill as a foundational formula for menopausal syndrome is well established, yet it may need to be combined with other herbs or therapies to maximize its therapeutic effect.

The decline in female reproductive function primarily manifests as diminished ovarian reserve and a poor ovarian response. Modern medicine suggests that this condition is closely associated with advanced age, ovarian failure, and decreased oocyte quality. In TCM theory, the pathogenesis of menopause is mainly attributed to kidney deficiency; particularly, it is emphasized that insufficient kidney essence leads to both kidney Qi and Yin deficiency (Han et al., 2023b). Patients commonly present with clinical manifestations such as infertility, menstrual disorders, soreness and weakness of the lower back and knees, dizziness, tinnitus, and fatigue. Studies have shown that kidney-tonifying and Yin-nourishing formulas, particularly EZTG, which is based on the Erzhi Pill, exert positive therapeutic effects on the decline of female reproductive function. In a randomized, double-blind, placebo-controlled trial, EZTG was observed to significantly improve embryo quality in advanced-age women undergoing *in vitro* fertilization, an effect attributed to the downregulation of apoptosis-related proteins in follicular fluid (Sun et al., 2021). Additionally, the results of another multicenter, randomized, double-blind, placebo-controlled trial have not yet been published (Han et al., 2023b). Furthermore, EZTG was found to enhance endometrial receptivity by upregulating DNA methyltransferase 1 protein expression, which improved the clinical pregnancy rate in infertile patients with kidney Yin deficiency (Fang et al., 2013). In another randomized trial evaluating the efficacy of EZTG intervention, the fertilization rate significantly increased (63.43% in the treatment group vs. 52.38% in the control group). Mechanistically, EZTG was reported to specifically regulate microRNAs related to follicular development, such as let-7e-5p, miR-140-3p, and miR-214-3p, in granulosa cells and influence signaling pathways, such as PI3K/Akt/mTOR, confirming its regulatory mechanism of “tonifying the kidneys, nourishing Yin, and promoting follicular development” at the molecular level (Liu et al., 2023). In summary, similar to its role in alleviating menopausal syndrome, although there is strong evidence supporting the use of Erzhi Pill for improving female reproductive function, the formulations studied are all modified and exclusively from the same research team. This once again reinforces the foundational role of Erzhi Pill in the treatment of reproductive system disorders.

Erzhi Pill and its modified formulations, either alone or in combination with other formulas, are frequently prescribed by TCM practitioners for treatment of diseases primarily attributed to the Liver–Kidney Yin deficiency pattern: gynecological disorders (e.g., threatened abortion and dysfunctional uterine bleeding), kidney diseases (e.g., IgA nephropathy and nephritis), and dermatological conditions (e.g., acne and seborrheic alopecia). Consistent with the TCM theory that “the kidneys govern bones,” Erzhi Pill also significantly ameliorated skeletal system disorders such as osteoporosis. However, rigorous randomized controlled trials are still required to validate the therapeutic effects derived from these clinical experiences. The details of the relevant TCM formulas are summarized in Supplementary Table S4.

In TCM, disease treatment is based on syndrome differentiation rather than pathophysiological manifestations. Consistently, Erzhi Pill is frequently included in formulas for diverse diseases. However, the precise application and efficacy of Erzhi are strongly influenced by the expertise of practitioners. By establishing quantifiable molecular indicators corresponding to TCM syndromes and linking these molecular markers to active metabolites of Erzhi Pill, a scientific basis for standardizing Erzhi Pill usage may be established.

## 7 Limitations and future perspectives

This work has presented the first comprehensive comparative analysis of Erzhi Pill and its constituent botanical drugs, Ligustri Lucidi Fructus and Ecliptae Herba, in which the phytochemical metabolites, quality control, pharmacological activity, and traditional and modern applications were evaluated. In addition, we briefly outlined the morphological and ecological characteristics of the constituent botanical drugs for an enriched understanding of Erzhi Pill. Analytical comparisons revealed that the Erzhi Pill formulation leverages the principles of pairing botanical drugs, with synergistic effects beyond simple additive outcomes. This synergy results in the development of novel metabolites, unique pharmacological actions, and distinct molecular mechanisms that are unattainable by the administration of individual botanical drugs separately. Thus, it provides a modern mechanistic corroboration for the traditional TCM theory of botanical drug pairing.

However, existing pharmacological and clinical studies on Erzhi Pill still have several limitations. At the basic research level, first, experimental designs often lack rigor, with most studies failing to include positive controls or dose gradients, and few have investigated gender differences in therapeutic response. Second, quality control information is frequently insufficient; although most studies provide botanical nomenclature, only a few of them implement strict quality control measures for raw materials and preparations. Third, the evidence hierarchy remains low as numerous *in vitro* and *in silico* findings are merely suggestive and lack *in vivo* validation. At the clinical research level, most indications lack high-quality evidence, which constitutes a primary obstacle hindering its internationalization. To address these limitations, future research should focus on the following aspects: first, standardizing experimental protocols by incorporating positive controls, multiple dose groups, and gender-specific analyses; second, strengthening quality control through strict adherence to the current *Chinese Pharmacopoeia* standards; third, establishing an integrated *in vitro*–*in vivo* evidence chain by validating molecular mechanisms through animal experiments, thereby constructing a complete evidence framework from molecular targets to whole-organism responses; and finally, conducting randomized, double-blind, placebo-controlled clinical trials focusing on advantageous indications such as menopausal syndrome and osteoporosis to confirm its clinical value with high-quality human evidence.

Classical botanical formulas in China are entering a unique development phase, with a “human-use experience” evaluation system that exempts clinical trials to significantly lower research and development barriers. Formulas such as Erzhi Pill have demonstrated substantial market potential. However, global recognition of the value of these formulas remains limited by their compositional complexity and regulatory disparities of different countries. To capitalize on this momentum, multidimensional innovations are imperative in several areas: 1) resource assurance, in which historical documentation, geo-information system, and DNA barcoding to authenticate *Ligustrum lucidum* and *Eclipta prostrata* origins are integrated to safeguard authentic cultivation; 2) process standardization, with unified preparation methods to reconcile efficacy differences between traditional and modern techniques; 3) quality control, in which bioactivity-aligned quality markers are established to bridge quality indicators and pharmacological substance bases; 4) mechanistic insights, with “drug-target co-screening” approaches used to decode the multiple metabolite, multitarget synergistic networks of Erzhi Pill; 5) safety evaluations, with enhanced good laboratory practice-compliant toxicology studies (acute, chronic, and reproductive toxicity) for international compliance; and 6) clinical repositioning, with precise indications defined via evidence-based approaches and biomarker validation. This systematic upgrading will drive a transition of Erzhi Pill from empirical application to scientifically driven modernization, providing substantial foundations for its secondary development and internationalized application.
