# Supplementary material for: The modern scientific interpretation of ancient wisdom: a review of the phytochemistry and pharmacology of Erzhi Pill and its constituent botanical drugs
Source: Front Pharmacol. 2026 Apr 21;17:1797126. doi: 10.3389/fphar.2026.1797126 (PMC13139087; doi:10.3389/fphar.2026.1797126)
Supplement: Supplementary file 1 [file Table1.docx]

**Table S1．Chemical compounds of LLF**

| Structure Class | Name | Ref. |
| --- | --- | --- |
| Iridoids | Specnuezhenide (Nuezhenide) | (He et al., 2001b) |
|  | Isonuezhenide | (He et al., 2001b) |
|  | 8(Z)-Nuezhenide A | (Huang et al., 2010) |
|  | Neonuezhenide | (He et al., 2001b) |
|  | Oleuropein | (He et al., 2001b) |
|  | Oleuropeinic acid | (Liu et al., 2021) |
|  | Oleuropein Aglycone | (Li et al., 2020) |
|  | Oleoside | (Li et al., 2020) |
|  | Oleoside dimethyl ester | (He et al., 2001b) |
|  | Ligustroside | (He et al., 2001b) |
|  | Lucidumosides A | (He et al., 2001b) |
|  | Lucidumosides B | (He et al., 2001b) |
|  | Lucidumosides C | (He et al., 2001a) |
|  | Lucidumosides D | (He et al., 2001a) |
|  | Nuezhenoside G13 | (Fu et al., 2010) |
|  | Oleoside 11-methyl ester | (Yang et al., 2010) |
|  | Oleoside-7-methyl ester | (Guo et al., 2011) |
|  | p-Hydroxyphenethyl 7-β-D-glucoside elenolic acid ester | (Yang et al., 2010) |
|  | 6′′′-acetylnicotiflorine | (Yang et al., 2010) |
|  | Nicotiflorine | (Yang et al., 2010) |
|  | Oleoside 7-ethyl 11-methyl ester | (Yang et al., 2010) |
|  | 6'-elenolylnicotiflorine | (Yang et al., 2010) |
|  | 4', 5'-(2'-Hydroxy ligustrosidic acid) dimer | (Guo, 2015) |
|  | 1′′′-O-β-D-glucosylformoside | (Huang et al., 2010) |
|  | Oleonuezhenide | (Fu et al., 2010) |
|  | Iso-oleonuezhenide | (Fu et al., 2010) |
|  | Elenolic acid | (Li et al., 2020) |
|  | Nuezhenidic acid | (Guo et al., 2011) |
|  | Ligustrosidic acid | (Guo et al., 2011) |
|  | Isoligustrosidic acid | (Aoki et al., 2012) |
|  | Loganic acid | (Li et al., 2020) |
|  | Ligustaloside A | (Guo et al., 2011) |
|  | Ligustaloside B | (Guo et al., 2011) |
|  | 10-hydroxyoleuropein | (Guo et al., 2011) |
|  | 10-hydroxyligustroside | (Guo et al., 2011) |
|  | Liguside A | (Huang et al., 2010) |
|  | Liguside B | (Huang et al., 2010) |
|  | 6′-O-trans-Cinnamoyl 8-Epikingisidic acid | (Aoki et al., 2012) |
|  | 6′-O-cis-cinnamoyl 8-Epikingisidic acid | (Aoki et al., 2012) |
|  | 6′-O-trans-cinnamoyl iso-8-Epikingisidic acid | (Zhang et al., 2013) |
|  | 3-O-cis-p-Coumaroyltormentic acid | (Li et al., 2020) |
|  | 3-O-cis-p-Coumaroyltormentic acid or  3-O-trans-p-Coumaroyltormentic acid | (Li et al., 2020) |
|  | 8-demethyl-7-ketoliganin | (Li et al., 2020) |
|  | Oleopolynuzhenide A | (Aoki et al., 2012) |
|  | Osmanthuside H | (Li et al., 2020) |
|  | Nuzhenal A | (Aoki et al., 2012) |
|  | Nuzhenal B | (Aoki et al., 2012) |
|  | Nuzhenal C | (Zhang et al., 2013) |
|  | Ligulucidumoside A | (Zhang et al., 2013) |
|  | Ligulucidumoside B | (Zhang et al., 2013) |
|  | Ligulucidumoside C | (Zhang et al., 2013) |
|  | Nuezhengalaside | (Ru; et al., 2014) |
|  | (3-ethylidene-2-oxotetrahydropyran-4-yl)-acetic acid methyl ester | (Liu et al., 2010) |
|  | Nuezhenelenoliciside | (Qiu et al., 2018) |
|  | Isojaslanceoside B | (Qiu et al., 2018) |
|  | 6′-O-trans-cinnamoyl-secologanoside | (Qiu et al., 2018) |
|  | Liguluciside A | (Pang et al., 2018) |
|  | Liguluciside B | (Pang et al., 2018) |
|  | Liguluciside C | (Pang et al., 2018) |
|  | Liguluciridoid A | (Pang et al., 2018) |
|  | Liguluciridoid B | (Pang et al., 2018) |
|  | Excelside B | (Pang et al., 2018) |
|  | 2″-epifraxamoside | (Pang et al., 2018) |
|  | Fraxamoside | (Pang et al., 2018) |
|  | Acetylnicotiflorine | (Li et al., 2020) |
|  | 10-hydroxyoleoside-7-dimethyl ester | (Guo et al., 2011) |
|  | 10-hydroxyoleoside-7-methyl ester | (Guo et al., 2011) |
|  | 10-hydroxyoleoside dimethyl ester | (Li et al., 2020) |
|  | Oleonin | (Huang et al., 2013) |
|  | Morroniside | (Ru; et al., 2014) |
|  | Dehydrologanin (Ketologanin) | (Ru; et al., 2014) |
|  | Syringin (Ligustrin) | (Ru; et al., 2014) |
|  | Methyloleoside 7-ethyl ester | (Fu et al., 2010) |
|  | Jaspolyside methyl ester | (Fu et al., 2010) |
| Triterpenoids | α-amyrin | (Guo et al., 2011) |
|  | β-amyrin | (Guo et al., 2011) |
|  | Betulin | (Li et al., 2020) |
|  | 2-hydroxyoleanic acid | (Guo et al., 2011) |
|  | 2-hydroxyursolic acid | (Guo et al., 2011) |
|  | 19-hydroxy-3-acetyl-ursolic acid | (Guo et al., 2011) |
|  | Oleanolic acid acetate | (Guo et al., 2011) |
|  | Ursolic acid acetate | (Guo et al., 2011) |
|  | Tormentic acid | (Guo et al., 2011) |
|  | Oleanolic acid | (Yim et al., 2001) |
|  | Ursolic acid | (Xia et al., 2011) |
|  | Acetyloleanolic acid | (Guo et al., 2011) |
|  | 19α-hydroxy-3-acetylursolic acid | (Li et al., 2020) |
|  | Acetylursolic acid | (Guo et al., 2011) |
|  | α-ursolic acid methyl ester | (Guo et al., 2011) |
|  | Sibiricose A3 | (Li et al., 2020) |
|  | Oleanolic acid β-D-glucopyranosyl ester | (Ru; et al., 2014) |
|  | Oleanolic acid deriv. | (Ru; et al., 2014) |
|  | Nuzhenidic acid | (Li et al., 2020) |
|  | Oleanolic acid-3-O-β-D-glucuronopyranoside | (Ru; et al., 2014) |
|  | 3β-O-acetylpomolic acid | (Li et al., 2020) |
|  | Kingiside | (Ru; et al., 2014) |
|  | 3β-O-trans-p-coumaroylmaslinicacid or 3β-O-cis-p-coumaroylmaslinicacid | (Li et al., 2020) |
|  | 3β-O-trans-p-coumaroylmaslinicacid or /3β-O-cis-p-coumaroylmaslinic acid | (Li et al., 2020) |
|  | (3a)-3-hydroxy-urs-12-en-28-oic acid, methyl ester | (Ru; et al., 2014) |
|  | Dammarenediol | (Tao et al., 2022) |
|  | 3β-acetyl-20S,24R-dammarane-25-ene-24-hydroperoxy-20-ol | (Xu et al., 2008) |
|  | 3β-(p-hydroxy-trans-cinnamoyloxy)-2α-hydroxy-oleanolic acid | (Ru; et al., 2014) |
|  | Olitoriside | (Ru; et al., 2014) |
|  | 20S,24R-dammarane-25-ene-24-hydroperoxy-3β,20-diol | (Xu et al., 2008) |
|  | 3β-acetyl-20S,25-epoxydammarane-24α-ol | (Xu et al., 2008) |
|  | 20S,25-epoxydammarane-3β,24α-diol | (Xu et al., 2008) |
|  | 20S-dammarane-23-ene-3β,20,25-triol | (Xu et al., 2008) |
| Phenylethanols | Salidroside | (Chen et al., 2013) |
|  | Hydroxytyrosol | (Chen et al., 2013) |
|  | Echinacoside | (He et al., 2018) |
|  | Acteoside (Verbascoside) | (Guo et al., 2011) |
|  | Isoverbascoside | (Li et al., 2020) |
|  | Tyrosol | (Chen et al., 2013) |
|  | Tyrosyl acetate | (Chen et al., 2013) |
|  | β-hydroxyverbascoside | (Li et al., 2020) |
|  | β-D-glucopyranoside, 2-(4-hydroxyphenethyl) 6-acetate | (Liu et al., 2010) |
|  | 3,4-dihydroxyphenethyl-β-D-glucoside | (Li et al., 2020) |
|  | 3,4-dihydroxyphenylethyl alcohol glucoside | (Ru; et al., 2014) |
|  | Neosyringalide | (Guo et al., 2011) |
|  | Phenethyl alcohol (PEL) | (Ru; et al., 2014) |
| Flavonoids | Luteolin | (Lu et al., 2023) |
|  | Quercetin | (Wu et al., 2016) |
|  | Apigenin | (Feng et al., 2019) |
|  | Quercetin 3-rutinoside (Rutin) | (Li et al., 2020) |
|  | Luteolin-7-O-glucoside | (Li et al., 2020) |
|  | Luteolin-7-O-rutinoside | (Li et al., 2020) |
|  | Kaempferol | (Ru; et al., 2014) |
|  | Taxifolin | (Ru; et al., 2014) |
|  | Mauritianin | (Ru; et al., 2014) |
|  | Baimaside (QUOSP) | (Ru; et al., 2014) |
|  | Hyperoside (Quercetin 3-galactoside) | (Li et al., 2020) |
|  | 10-hydroxy ligustroflavone | (Li et al., 2020) |
|  | Nuezhenoside ([Ligustroflavone](https://pubchem.ncbi.nlm.nih.gov/compound/10417462)) | (Liu et al., 2010) |
|  | Quercetin-3-O-rhamnoglucoside | (Ru; et al., 2014) |
|  | Apigetrin (Cosmetin; Apigenin 7-glucoside) | (Ru; et al., 2014) |
|  | Kaempferol-3-O-α-L-rhamnosyl(1→2)-β-D-glucoside | (Ru; et al., 2014) |
|  | Eriodictyol | (Ru; et al., 2014) |
|  | Manghaslin | (Ru; et al., 2014) |
|  | Clitorin | (Ru; et al., 2014) |
|  | Daidzein | (Ru; et al., 2014) |
| Monoterpenoids excluding iridoids | (-)-α-pinene | (Ru; et al., 2014) |
|  | (-)-β-pinene | (Ru; et al., 2014) |
|  | L-bornyl acetate | (Ru; et al., 2014) |
|  | (R)-linalool | (Ru; et al., 2014) |
|  | L-Limonene | (Ru; et al., 2014) |
|  | Borneol / Linderol | (Ru; et al., 2014) |
|  | Vomifoliol | (Ru; et al., 2014) |
|  | Linalyl acetate | (Ru; et al., 2014) |
|  | Nerol | (Ru; et al., 2014) |
|  | Geraniol | (Ru; et al., 2014) |
|  | 4-terpineol | (Ru; et al., 2014) |
|  | (4S)-4-hydroxy-3,5,5-trimethyl-4-[(E,3R)-3-[(2R,3R,4S,5S,6R)-3,4,5-trihydroxy-6-(hydroxymethyl)tetrahydropyran-2-yl]oxybut-1-enyl]cyclohex-2-en-1-one | (Ru; et al., 2014) |
| Sesquiterpenoids | Thujopsene | (Ru; et al., 2014) |
|  | α-humulene | (Ru; et al., 2014) |
|  | Cedrol | (Ru; et al., 2014) |
|  | Damascenone | (Ru; et al., 2014) |
| Phenylpropanoids | Sinapyl alcohol | (Ru; et al., 2014) |
|  | Chlorogenic acid | (Seo et al., 2017) |
|  | Caffeic acid | (Ru; et al., 2014) |
|  | Protocatechuic acid | (Li et al., 2020) |
|  | Protocatechuic Aldehyde | (Li et al., 2020) |
|  | Methylcinnamate | (Ru; et al., 2014) |
|  | Eugenol | (Ru; et al., 2014) |
|  | Coniferin | (Ru; et al., 2014) |
|  | Olivil | (Ru; et al., 2014) |
|  | Dihydrosyringin | (Ru; et al., 2014) |
|  | Liriodendrin (Syringaresinol diglucoside) | (Ru; et al., 2014) |
|  | Dihydrosinapyl alcohol | (Ru; et al., 2014) |
|  | Olivil-4'-O-β-d-glucopyranoside | (Ru; et al., 2014) |
|  | Coniferol | (Ru; et al., 2014) |
| Steroids | Sitogluside | (Ru; et al., 2014) |
|  | β-sitosterol | (Ru; et al., 2014) |
|  | (20S)-24-ene-3β,20-diol-3-acetate | (Ru; et al., 2014) |
| Carbohydrates | D-Mannoheptulose | (Ru; et al., 2014) |
|  | Mannite/Mannitol | (Ru; et al., 2014) |
|  | Sucrose | (Ru; et al., 2014) |
|  | α-L-Rhamnose | (Ru; et al., 2014) |
| Phenolics | Urushiol III | (Ru; et al., 2014) |
|  | Pyrocatechol (1,2-Dihydroxybenzene) | (Li et al., 2020) |
| Organic acids | Quinic acid | (Li et al., 2020) |
| Esters | Butyl butyryllactate | (Ru; et al., 2014) |
|  | Dibutyl phthalate | (Ru; et al., 2014) |
|  | delta-valerolactone | (Li et al., 2020) |
| Anthraquinone | Physcion | (Li et al., 2020) |
| Alkaloids | Lucidusculine | (Ru; et al., 2014) |
|  | Nuphleine | (Ru; et al., 2014) |
|  | Cimidahurinine | (Ru; et al., 2014) |
|  | Spectabiline | (Ru; et al., 2014) |

Owing to methodological constraints, the determination of whether certain compounds qualify as isomers remains challenging.

**Reference**

AOKI, S., HONDA, Y., KIKUCHI, T., MIURA, T., SUGAWARA, R., YAOITA, Y., KIKUCHI, M. & MACHIDA, K. (2012). Six new secoiridoids from the dried fruits of Ligustrum lucidum. *Chem Pharm Bull (Tokyo),* 60**,** 251-6. doi:10.1248/cpb.60.251

CHEN, Q., YANG, L., ZHANG, G. & WANG, F. (2013). Bioactivity-guided Isolation of antiosteoporotic compounds from Ligustrum lucidum. *Phytother Res,* 27**,** 973-9. doi:10.1002/ptr.4820

FENG, R., DING, F., MI, X. H., LIU, S. F., JIANG, A. L., LIU, B. H., LIAN, Y., SHI, Q., WANG, Y. J. & ZHANG, Y. (2019). Protective Effects of Ligustroflavone, an Active Compound from Ligustrum lucidum, on Diabetes-Induced Osteoporosis in Mice: A Potential Candidate as Calcium-Sensing Receptor Antagonist. *Am J Chin Med,* 47**,** 457-476. doi:10.1142/s0192415x1950023x

FU, G., IP, F. C., PANG, H. & IP, N. Y. (2010). New secoiridoid glucosides from Ligustrum lucidum induce ERK and CREB phosphorylation in cultured cortical neurons. *Planta Med,* 76**,** 998-1003. doi:10.1055/s-0029-1240869

GUO, L. G. X. L. C. L. Z. W. T. (2015). A rare secoiridoid dimer derivative from Ligustri lucidi fructus. *Records of Natural Products,* Vol.3**,** 323-328.

GUO, N., YU, Y., ABLAJAN, K., LI, L., FAN, B., PENG, J., YAN, H., MA, F. & NIE, Y. (2011). Seasonal variations in metabolite profiling of the fruits of Ligustrum lucidum Ait. *Rapid Commun Mass Spectrom,* 25**,** 1701-14. doi:10.1002/rcm.5036

HE, F., CHEN, L., LIU, Q., WANG, X., LI, J. & YU, J. (2018). Preparative Separation of Phenylethanoid and Secoiridoid Glycosides from Ligustri Lucidi Fructus by High-Speed Counter-Current Chromatography Coupled with Ultrahigh Pressure Extraction. *Molecules,* 23. doi:10.3390/molecules23123353

HE, Z. D., BUT, P. P. H., CHAN, T. W., DONG, H., XU, H. X., LAU, C. P. & SUN, H. D. (2001a). Antioxidative glucosides from the fruits of Ligustrum lucidum. *Chem Pharm Bull (Tokyo),* 49**,** 780-4. doi:10.1248/cpb.49.780

HE, Z. D., DONG, H., XU, H. X., YE, W. C., SUN, H. D. & BUT, P. P. (2001b). Secoiridoid constituents from the fruits of Ligustrum lucidum. *Phytochemistry,* 56**,** 327-30. doi:10.1016/s0031-9422(00)00406-4

HUANG, X.-J., WANG, L., SHAO, M., HU, S.-Z., JIANG, R.-W., YAO, X.-S., LI, Y.-L., YIN, Z.-Q., WANG, Y. & YE, W.-C. (2013). Oleonin, the first secoiridoid with 1α-configuration from Ligustrum lucidum. *RSC Advances,* 3. doi:10.1039/c3ra40557e

HUANG, X. J., WANG, Y., YIN, Z. Q. & YE, W. C. (2010). Two new dimeric secoiridoid glycosides from the fruits of Ligustrum lucidum. *J Asian Nat Prod Res,* 12**,** 685-90. doi:10.1080/10286020.2010.490781

LI, M., WANG, X., HAN, L., JIA, L., LIU, E., LI, Z., YU, H., WANG, Y., GAO, X. & YANG, W. (2020). Integration of multicomponent characterization, untargeted metabolomics and mass spectrometry imaging to unveil the holistic chemical transformations and key markers associated with wine steaming of Ligustri Lucidi Fructus. *J Chromatogr A,* 1624**,** 461228. doi:10.1016/j.chroma.2020.461228

LIU, J., LIU, Z., WANG, L., HE, H., MU, H., SUN, W., ZHOU, Y., LIU, Y., MA, W., ZHANG, W., FU, M., FAN, Y. & SONG, X. (2021). Bioactivity-guided isolation of immunomodulatory compounds from the fruits of Ligustrum lucidum. *J Ethnopharmacol,* 274**,** 114079. doi:10.1016/j.jep.2021.114079

LIU, X., WANG, C.-Y., SHAO, C.-L., FANG, Y.-C., WEI, Y.-X., ZHENG, C.-J., SUN, L.-L. & GUAN, H.-S. (2010). Chemical constituents from the fruits of Ligustrum lucidum. *Chemistry of Natural Compounds,* 46**,** 701-703. doi:10.1007/s10600-010-9719-x

LU, Z., GAO, F., TENG, F., TIAN, X., GUAN, H., LI, J., WANG, X., LIANG, J., TIAN, Q. & WANG, J. (2023). Exploring the pathogenesis of depression and potential antidepressants through the integration of reverse network pharmacology, molecular docking, and molecular dynamics. *Medicine (Baltimore),* 102**,** e35793. doi:10.1097/md.0000000000035793

PANG, X., ZHAO, J. Y., YU, H. Y., YU, L. Y., WANG, T., ZHANG, Y., GAO, X. M. & HAN, L. F. (2018). Secoiridoid analogues from the fruits of Ligustrum lucidum and their inhibitory activities against influenza A virus. *Bioorg Med Chem Lett,* 28**,** 1516-1519. doi:10.1016/j.bmcl.2018.03.080

QIU, Z. C., ZHAO, X. X., WU, Q. C., FU, J. W., DAI, Y., WONG, M. S. & YAO, X. S. (2018). New secoiridoids from the fruits of Ligustrum lucidum. *J Asian Nat Prod Res,* 20**,** 431-438. doi:10.1080/10286020.2018.1454438

RU;, J., LI;, P., WANG;, J., ZHOU;, W., LI;, B., HUANG;, C., LI;, P., GUO;, Z., TAO;, W., YANG;, Y., XU;, X., LI;, Y., WANG;, Y. & YANG, L. (2014). TCMSP: a database of systems pharmacology for drug discovery from herbal medicines. *J Cheminformatics,* 6**,** 13. doi:10.1186/1758-2946-6-13

SEO, H. L., BAEK, S. Y., LEE, E. H., LEE, J. H., LEE, S. G., KIM, K. Y., JANG, M. H., PARK, M. H., KIM, J. H., KIM, K. J., LEE, H. S., AHN, S. C., LEE, J. R., PARK, S. J., KIM, S. C. & KIM, Y. W. (2017). Liqustri lucidi Fructus inhibits hepatic injury and functions as an antioxidant by activation of AMP-activated protein kinase in vivo and in vitro. *Chem Biol Interact,* 262**,** 57-68. doi:10.1016/j.cbi.2016.11.031

TAO, R., LIU, E., ZHAO, X., HAN, L., YU, B., MAO, H., YANG, W. & GAO, X. (2022). Combination of Ligustri Lucidi Fructus with Ecliptae Herba and their phytoestrogen or phytoandrogen like active pharmaceutical ingredients alleviate oestrogen/testosterone-induced benign prostatic hyperplasia through regulating steroid 5-α-reductase. *Phytomedicine,* 102**,** 154169. doi:10.1016/j.phymed.2022.154169

WU, Y., LI, Q., LI, X., HE, D., NIU, M. U., LU, X. & LI, H. (2016). Effect of the Fructus Ligustri Lucidi extract and its monomers quercetin and oleanolic acid on the adhesion and migration of melanocytes and intracellular actin. *Biomed Rep,* 4**,** 583-588. doi:10.3892/br.2016.638

XIA, E. Q., WANG, B. W., XU, X. R., ZHU, L., SONG, Y. & LI, H. B. (2011). Microwave-assisted extraction of oleanolic acid and ursolic acid from Ligustrum lucidum Ait. *Int J Mol Sci,* 12**,** 5319-29. doi:10.3390/ijms12085319

XU, X. H., YANG, N. Y., QIAN, S. H., XIE, N. & DUAN, J. A. (2008). Dammarane triterpenes from Ligustrum lucidum. *J Asian Nat Prod Res,* 10**,** 33-7. doi:10.1080/10286020701273833

YANG, N.-Y., XU, X.-H., REN, D.-C., DUAN, J.-A., XIE, N., TIAN, L.-J. & QIAN, S.-H. (2010). Secoiridoid Constituents from the Fruits ofLigustrum lucidum. *Helvetica Chimica Acta,* 93**,** 65-71. doi:10.1002/hlca.200900144

YIM, T. K., WU, W. K., PAK, W. F. & KO, K. M. (2001). Hepatoprotective action of an oleanolic acid-enriched extract of Ligustrum lucidum fruits is mediated through an enhancement on hepatic glutathione regeneration capacity in mice. *Phytother Res,* 15**,** 589-92. doi:10.1002/ptr.878

ZHANG, Y., LIU, L., GAO, J., WU, C., HAN, L., LIU, E., SHI, P., GAO, X. & WANG, T. (2013). New secoiridoids from the fruits of Ligustrum lucidum Ait with triglyceride accumulation inhibitory effects. *Fitoterapia,* 91**,** 107-112. doi:10.1016/j.fitote.2013.08.022
