# Supplementary figures and images for: The modern scientific interpretation of ancient wisdom: a review of the phytochemistry and pharmacology of Erzhi Pill and its constituent botanical drugs
Source: Front Pharmacol. 2026 Apr 21;17:1797126. doi: 10.3389/fphar.2026.1797126 (PMC13139087; doi:10.3389/fphar.2026.1797126)

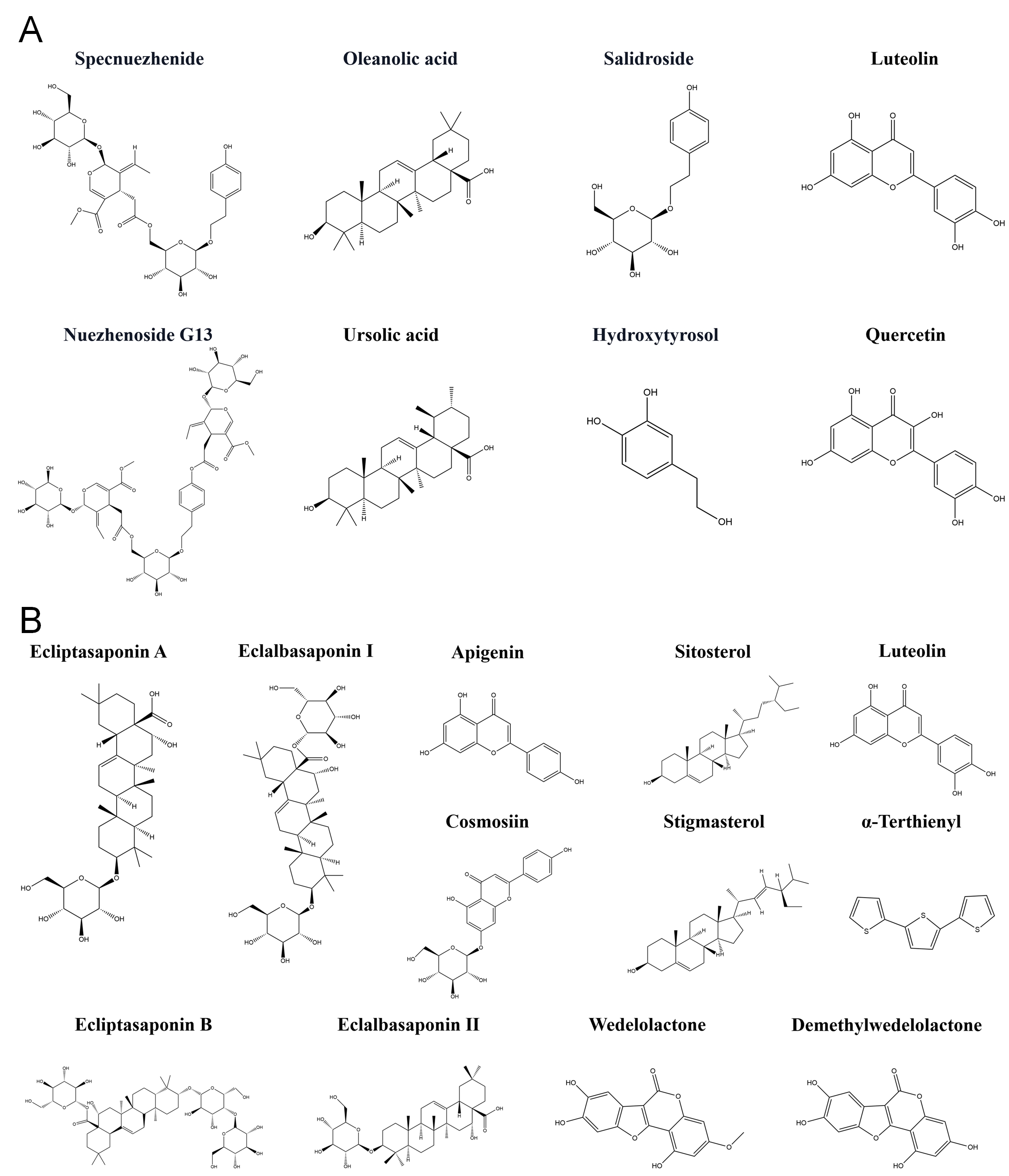

Supplement: Supplementary file 2 [file Image1.jpeg]
