# Supplementary material for: The modern scientific interpretation of ancient wisdom: a review of the phytochemistry and pharmacology of Erzhi Pill and its constituent botanical drugs
Source: Front Pharmacol. 2026 Apr 21;17:1797126. doi: 10.3389/fphar.2026.1797126 (PMC13139087; doi:10.3389/fphar.2026.1797126)
Supplement: Supplementary file 3 [file Table2.docx]

**Table S2．Chemical constituents of EH**

| Structure Class | Name | Ref. |
| --- | --- | --- |
| Triterpenoids | Ecliptasaponin A | (Sun et al., 2010) |
|  | Ecliptasaponin B | (Ru; et al., 2014) |
|  | Ecliptasaponin C | (Han et al., 2015) |
|  | Ecliptasaponin D | (Ru; et al., 2014) |
|  | Eclalbasaponins I | (Xi et al., 2014a) |
|  | Eclalbasaponins Ⅱ | (Xi et al., 2014a) |
|  | Eclalbasaponins Ⅲ | (Xi et al., 2014a) |
|  | Eclalbasaponins Ⅳ | (Kim et al., 2015) |
|  | Eclalbasaponins Ⅴ | (Le et al., 2021) |
|  | Eclalbasaponins VI | (Xi et al., 2014a) |
|  | Eclalbasaponins VII | (Kim et al., 2015) |
|  | Eclalbasaponins VIII | (Xi et al., 2014a) |
|  | Silphioside B | (Xi et al., 2014a) |
|  | Silphioside E | (Xi et al., 2014a) |
|  | Echinocystic acid | (Han et al., 2013) |
|  | β-amyrone | (Sun et al., 2010) |
|  | Oleanolic acid | (Sun et al., 2010) |
|  | Ursolic acid | (Li et al., 2023) |
|  | Amyrin | (Ru; et al., 2014) |
|  | Machaeroceric acid | (Le et al., 2021) |
|  | Echinocystic acid-3-O-(6-O-acetyl)- β-D-glucopyranoside | (Kim et al., 2015) |
|  | Echinocystic acid-28-O-β-D-glucopyranoside | (Han et al., 2013) |
|  | 28-O-β-d-glucopyranosyl betulinic acid 3β-O-β-d-glucopyranoside | (Xi et al., 2014a) |
|  | 3-oxo-16a-hydroxy-olean-12-en-28-oic acid | (Han et al., 2013) |
|  | 3β,16β,29-trihydroxy oleanane-12-ene-3-O-β-D-glucopyranoside | (Xi et al., 2014a) |
|  | 3,28-di-O-β-D-glucopyranosyl-3β,16β-dihydroxy oleanane-12-ene-28-oleanlic acid | (Xi et al., 2014a) |
|  | 3-O-β-D-glucopyranosyl-(1→2)-β-D-glucopyranosyl oleanlic-18ene acid-28-O-β-D-glucopyranoside | (Xi et al., 2014a) |
|  | 3-O-(2-O-acetyl-β-D-glucopyranosyl) oleanolic acid-28-O-(β-D-glucopyranosyl) ester | (Xi et al., 2014b) |
|  | 3-O-(6-O-acetyl-β-D-glucopyranosyl) oleanolic acid-28-O-(β-D-glucopyranosyl) ester | (Xi et al., 2014b) |
|  | 3-O-(β-D-glucopyranosyl) oleanolic acid-28-O-(6-O-acetyl-β-D-glucopyranosyl) ester | (Xi et al., 2014b) |
| Flavonoids | Luteolin | (Kim et al., 2015) |
|  | Pratensein | (Kim et al., 2015) |
|  | Apigenin | (Kim et al., 2015) |
|  | Linarin | (Li et al., 2023) |
|  | Acacetin | (Ru; et al., 2014) |
|  | Butin | (Li et al., 2023) |
|  | Myricetin | (Li et al., 2023) |
|  | Luteolin sulfate | (Han et al., 2015) |
|  | Apigenin sulfate | (Han et al., 2015) |
|  | Apigenin 7-glucoside/ Cosmetin | (Ru; et al., 2014) |
|  | Pratensein-7-O-β-D-glucopyranoside | (Kim et al., 2015) |
|  | 7-O-methylorobol-4′-O-β-D-glucopyranoside | (Han et al., 2013) |
|  | Quercetin | (Sun et al., 2010) |
|  | Diosmetin | (Lee et al., 2009) |
|  | Astragalin (Kaempferol 3-glucoside) | (Li et al., 2023) |
|  | 3′-hydroxybiochanin A | (Lee et al., 2009) |
|  | 3′-O-methylorobol | (Lee et al., 2009) |
|  | Acacetin 7-rutinoside | (Jeong, 2013) |
|  | Apigenin-7-glucuronide | (Li et al., 2023) |
|  | Sedelolactone | (Jeong, 2013) |
|  | Tricetin | (Le et al., 2021) |
|  | Diosmetin-7-O-beta-D-glucopyranoside | (Li et al., 2023) |
|  | Luteolin-7-O-β-D-glucoside | (Han et al., 2015) |
|  | Kaemferol-7-O-α-D-rhamnoside | (Le et al., 2021) |
|  | Quercetin-3-O-β-D-glucoside | (Le et al., 2021) |
|  | Hesperetin-7-O-β-D-glucoside | (Le et al., 2021) |
|  | Orobol | (Le et al., 2021) |
|  | Cyanidin 3-O-glucoside | (Li et al., 2023) |
|  | (Z)-1-(2,4-dihydroxyphenyl)-3-(3,4-dihydroxyphenyl)prop-2-en-1-one | (Ru; et al., 2014) |
| Coumarins | Wedelolactone | (Sun et al., 2010) |
|  | Demethylwedelolactone | (Sun et al., 2010) |
|  | Coumestan | (Ru; et al., 2014) |
|  | Fraxetin | (Li et al., 2023) |
|  | Demethylwedelolactone-7-glucoside | (Ru; et al., 2014) |
|  | 1,3,8,9-tetrahydroxycoumestan 3-sulfate | (Giang et al., 2024) |
| Phenolics | merulinic acid C | (Sun et al., 2010) |
|  | Mequinol | (Jeong, 2013) |
| Thiophenes | α-terthienyl | (Kim et al., 2015) |
|  | α-terthienylmethanol | (Xi et al., 2014a) |
|  | α-formylterthienyl | (Xi et al., 2014a) |
|  | Ecliptal | (Kim et al., 2015) |
|  | α-Terthienyl methyl acetate | (Ru; et al., 2014) |
|  | 3′-methoxy-2,2′:5′,2′′-terthiophene | (Kim et al., 2015) |
|  | 5-(3′′,4′′-dihydroxy-1′′-butynyl)-2,2′-bithiophene | (Kim et al., 2015) |
|  | 5-(but-3-yne-1,2-diol)-50-hydroxy-methyl-2,20-bithiophene | (Xi et al., 2014a) |
|  | 2-(penta-1,3-diynyl)-5-(3,4-dihydroxy-but-1-ynyl)-thiophene | (Xi et al., 2014a) |
|  | 5-methoxymethyl-2,2′:5′,2″-terthiophene | (Xi et al., 2014a) |
|  | 5-ethoxymethyl-2,2′:5′,2″-terthiophene | (Xi et al., 2014a) |
|  | 5-(4-chloro-3-hydroxybut-1-ynyl) thiophene | (Giang et al., 2024) |
|  | 2-(Buta-1,3-diynyl)-5-(4-chloro-3-hydroxybut-1-ynyl) thiophene | (Ru; et al., 2014) |
|  | (2,2':5',2''-Terthiophene)-5-carboxylic acid | (Ru; et al., 2014) |
| Alkaloids | 20-epi-3-dehydroxy-3-oxo-5,6-dihydro-4,5-dehydroverazine | (Abdel-Kader et al., 1998) |
|  | Verazine | (Abdel-Kader et al., 1998) |
|  | Ecliptalbine | (Abdel-Kader et al., 1998) |
|  | (20R)-4β-hydroxyverazine | (Abdel-Kader et al., 1998) |
|  | (20R)-25β-hydroxyverazine | (Abdel-Kader et al., 1998) |
|  | (20R) epimer of verazine | (Abdel-Kader et al., 1998) |
|  | 4β-hydroxyverazine | (Abdel-Kader et al., 1998) |
|  | 25β-hydroxyverazine | (Abdel-Kader et al., 1998) |
|  | Ecliptine | (Ameen et al., 2023) |
|  | Nicotine | (Ru; et al., 2014) |
|  | Demissine | (Ru; et al., 2014) |
|  | 3-[(2S)-2,3-dihydroxy-3-methyl-butyl]-4-methoxy-1-methyl-carbostyril | (Ru; et al., 2014) |
| Polyacetylenes | (5E)-hendeca-1,5dien-7,9-diyne-diol-4-O-β-D-glucopyranoside | (Meng et al., 2019) |
|  | Eprostrata Ⅰ | (Meng et al., 2019) |
|  | (5E)-trideca-1,5-dien-7,9,11-triyne-3,4-diol-4-O-β-D-glucopyranoside | (Xi et al., 2014a) |
|  | 3-O-β-D-glucopyranosyloxy-1-hydroxy-4E,6E-tetradecene-8,10,12-triyne | (Xi et al., 2014a) |
|  | 2-O-β-D-glucosyltrideca-3E,11E-dien5,7,9-triyne-1,2,13-triol | (Meng et al., 2019) |
|  | 2-O-β-D-glucosyltrideca-3E,11E-dien-5,7,9-triyne-1,2-diol | (Meng et al., 2019) |
|  | 2-O-β-D-glucosyltrideca-3E,11Z-dien-5,7,9triyne–1,2-diol | (Meng et al., 2019) |
| Monoterpenoids | rel-(1S,2S,3S,4R,6R)-1,6-epoxy-menthane-2,3-diol-3-O-β-d-glucopyranoside | (Xi et al., 2014a) |
|  | rel-(1S,2S,3S,4R,6R)-3-O-(6-O-caffeoyl-β-d-glucopyranosyl)-1,6-epoxy menthane-2,3-diol | (Xi et al., 2014a) |
|  | (2E,6E)-2,6,10-trimethyl-2,6,11-dodecatriene-1,10-diol-1-O-β-d-glucopyranoside | (Xi et al., 2014a) |
|  | (1R,2R,4R)-Dihydrocarveol | (Ru; et al., 2014) |
| Sesquiterpenoids | (-)-Caryophyllene oxide | (Ru; et al., 2014) |
|  | Abscisic acid | (Li et al., 2023) |
| Benzoic acids | ethyl 2,6-dihydroxy-4-methoxybenzoate | (Sun et al., 2010) |
|  | 3,5-dihydroxybenzoic acid | (Li et al., 2023) |
|  | 3,4-dihydroxybenzoic acid ethyl ester | (Han et al., 2015) |
|  | 4-hydroxybenzoic acid | (Le et al., 2021) |
|  | Syringic acid | (Le et al., 2021) |
|  | Vanillic acid | (Jeong, 2013) |
| Steroids | Stigmasterol glucoside | (Le et al., 2021) |
|  | Stigmasterol | (Melo et al., 1994) |
|  | β-Sitosterol | (Melo et al., 1994) |
|  | Caulophyllogenin | (Ru; et al., 2014) |
|  | Chloromaloside | (Ru; et al., 2014) |
|  | (3S,8S,9S,10R,13R,14S,17R)-17-[(1R,4R)-1,4-dimethylhexyl]-10,13-dimethyl-2,3,4,7,8,9,11,12,14,15,16,17-dodecahydro-1H-cyclopenta[a]phenanthren-3-ol | (Ru; et al., 2014) |
|  | (3S,8S,9S,10R,13R,14S,17R)-17-[(E,1R,4R)-1,4-dimethylhex-2-enyl]-10,13-dimethyl-2,3,4,7,8,9,11,12,14,15,16,17-dodecahydro-1H-cyclopenta[a]phenanthren-3-ol | (Ru; et al., 2014) |
| Phenylpropanoids  excluding Coumarins | Caffeic acid | (Li et al., 2023) |
|  | 1,4-Dicaffeoylquinic acid | (Li et al., 2023) |
|  | 1-Caffeoylquinic acid | (Li et al., 2023) |
|  | Chlorogenic acid | (Le et al., 2021) |
|  | Protocatechuic acid | (Le et al., 2021) |
|  | Ferulic acid | (Jeong, 2013) |
| Esters | Isodesacetyluvaricin | (Ru; et al., 2014) |
|  | senecioester | (Giang et al., 2024) |
|  | tiglinsaureester | (Giang et al., 2024) |
| Carbohydrates | (2S)-1-O-stearoyl-3-O-β-D-galactopyranosyl-snglycerol | (Le et al., 2021) |
|  | (2S)-3-O-(9Z,12Z-octadecadienoyl)-glyceryl-O-β-D-galactopyranoside | (Le et al., 2021) |
| Hydroxyaldehydes | 4-hydroxyaldehyde | (Jeong, 2013) |
| Carboxylic acids | Nicotinic acid | (Ru; et al., 2014) |
| Hydrocarbons | (1S,4S)-7-isopropylidene-1,4-dimethyl-2,3,4,5,6,8-hexahydro-1H-azulene | (Ru; et al., 2014) |
| Amino Acid | L-Asparagine | (Li et al., 2023) |

Owing to methodological constraints, the determination of whether certain compounds qualify as isomers remains challenging. And as the medicinal part of Ecliptae Herba is the above-ground portion, cited literatures all use it or its components (e.g., stems and leaves) as research material.

**Reference**

ABDEL-KADER, M. S., BAHLER, B. D., MALONE, S., WERKHOVEN, M. C., VAN TROON, F., DAVID, WISSE, J. H., BURSUKER, I., NEDDERMANN, K. M., MAMBER, S. W. & KINGSTON, D. G. (1998). DNA-damaging steroidal alkaloids from Eclipta alba from the suriname rainforest1. *J Nat Prod,* 61**,** 1202-8. doi:10.1021/np970561c

AMEEN, F., ORFALI, R., MAMIDALA, E. & DAVELLA, R. (2023). In silico toxicity prediction, molecular docking studies and in vitro validation of antibacterial potential of alkaloids from Eclipta alba in designing of novel antimicrobial therapeutic strategies. *Biotechnol Genet Eng Rev,* 39**,** 760-774. doi:10.1080/02648725.2022.2162264

GIANG, L. T., PARK, S., CUC, N. T., TAI, B. H., KIEM, P. V., HANG, N. T. M., BAN, N. K., CUONG, P. V. & NHIEM, N. X. (2024). Bithiophene and coumestan derivatives from Eclipta prostrata (L.) L. and their hepatoprotective activity. *J Asian Nat Prod Res,* 26**,** 1381-1387. doi:10.1080/10286020.2024.2364912

HAN, L.-F., ZHAO, J., ZHANG, Y., KOJO, A., LIU, E.-W. & WANG, T. (2013). Chemical Constituents from Dried Aerial Parts of Eclipta prostrata. *Chinese Herbal Medicines,* 5**,** 313-316. doi:10.1016/S1674-6384(13)60047-7

HAN, L., LIU, E., KOJO, A., ZHAO, J., LI, W., ZHANG, Y., WANG, T. & GAO, X. (2015). Qualitative and quantitative analysis of Eclipta prostrata L. by LC/MS. *ScientificWorldJournal,* 2015**,** 980890. doi:10.1155/2015/980890

JEONG, E. J. L., M.K.;KIM, Y.C.;SUNG, S.H. (2013). Antiproliferative Phenolics from Eclipta prostrata in the Activated Hepatic Stellate Cells. *Natural Product Sciences,* 19**,** 231-235.

KIM, H. Y., KIM, H. M., RYU, B., LEE, J. S., CHOI, J. H. & JANG, D. S. (2015). Constituents of the aerial parts of Eclipta prostrata and their cytotoxicity on human ovarian cancer cells in vitro. *Arch Pharm Res,* 38**,** 1963-9. doi:10.1007/s12272-015-0599-2

LE, D. D., NGUYEN, D. H., MA, E. S., LEE, J. H., MIN, B. S., CHOI, J. S. & WOO, M. H. (2021). PTP1B Inhibitory and Anti-inflammatory Properties of Constituents from Eclipta prostrata L. *Biol Pharm Bull,* 44**,** 298-304. doi:10.1248/bpb.b20-00994

LEE, M. K., HA, N. R., YANG, H., SUNG, S. H. & KIM, Y. C. (2009). Stimulatory constituents of Eclipta prostrata on mouse osteoblast differentiation. *Phytother Res,* 23**,** 129-31. doi:10.1002/ptr.2560

LI, H., SHI, W., SHEN, T., HUI, S., HOU, M., WEI, Z., QIN, S., BAI, Z. & CAO, J. (2023). Network pharmacology-based strategy for predicting therapy targets of Ecliptae Herba on breast cancer. *Medicine (Baltimore),* 102**,** e35384. doi:10.1097/md.0000000000035384

MELO, P. A., DO NASCIMENTO, M. C., MORS, W. B. & SUAREZ-KURTZ, G. (1994). Inhibition of the myotoxic and hemorrhagic activities of crotalid venoms by Eclipta prostrata (Asteraceae) extracts and constituents. *Toxicon,* 32**,** 595-603. doi:10.1016/0041-0101(94)90207-0

MENG, X., LI, B. B., LIN, X., JIANG, Y. Y., ZHANG, L., LI, H. Z. & CUI, L. (2019). New polyacetylenes glycoside from Eclipta prostrate with DGAT inhibitory activity. *J Asian Nat Prod Res,* 21**,** 501-506. doi:10.1080/10286020.2018.1452914

RU;, J., LI;, P., WANG;, J., ZHOU;, W., LI;, B., HUANG;, C., LI;, P., GUO;, Z., TAO;, W., YANG;, Y., XU;, X., LI;, Y., WANG;, Y. & YANG, L. (2014). TCMSP: a database of systems pharmacology for drug discovery from herbal medicines. *J Cheminformatics,* 6**,** 13. doi:10.1186/1758-2946-6-13

SUN, Z.-H., ZHANG, C.-F. & ZHANG, M. (2010). A New Benzoic Acid Derivative from Eclipta prostrata. *Chinese Journal of Natural Medicines,* 8**,** 244-246. doi:10.1016/S1875-5364(10)60033-7

XI, F. M., LI, C. T., HAN, J., YU, S. S., WU, Z. J. & CHEN, W. S. (2014a). Thiophenes, polyacetylenes and terpenes from the aerial parts of Eclipata prostrate. *Bioorg Med Chem,* 22**,** 6515-22. doi:10.1016/j.bmc.2014.06.051

XI, F. M., LI, C. T., MI, J. L., WU, Z. J. & CHEN, W. S. (2014b). Three new olean-type triterpenoid saponins from aerial parts of Eclipta prostrata (L.). *Nat Prod Res,* 28**,** 35-40. doi:10.1080/14786419.2013.832674
