# Supplementary material for: The modern scientific interpretation of ancient wisdom: a review of the phytochemistry and pharmacology of Erzhi Pill and its constituent botanical drugs
Source: Front Pharmacol. 2026 Apr 21;17:1797126. doi: 10.3389/fphar.2026.1797126 (PMC13139087; doi:10.3389/fphar.2026.1797126)
Supplement: Supplementary file 4 [file Table3.docx]

**Table S3．Chemical compounds have been isolated and identified from EZW**

| Structure Class | Source | Chemical compounds | Ref. |
| --- | --- | --- | --- |
| Iridoids | LLF | Loganic acid | (Jia et al., 2018) |
|  | LLF | Nuezhenidic acid | (Jia et al., 2018) |
|  | LLF | Elenolic acid | (Jia et al., 2018) |
|  | LLF | Nuzhenal A | (Jia et al., 2018) |
|  | LLF | Nuzhenal C | (Zhu et al., 2023) |
|  | LLF | 10-hydroxyoleoside dimethylester | (Jia et al., 2018) |
|  | LLF | Oleoside (Secologanoside) | (Jia et al., 2018) |
|  | NEW | Sweroside | (Jia et al., 2018) |
|  | LLF | Nuezhenoside G13 | (Zhu et al., 2023) |
|  | LLF | Oleuropein aglycone | (Zhu et al., 2023) |
|  | LLF | Oleuropein aglycone or isomer | (Jia et al., 2018) |
|  | NEW | Secologanol | (Jia et al., 2018) |
|  | LLF | Ligulucidumoside A | (Jia et al., 2018) |
|  | LLF | Ligulucidumoside C | (Jia et al., 2018) |
|  | LLF | Oleoside 11-methyl ester | (Zhu et al., 2023) |
|  | NEW | Secoxyloganin methyl ester | (Jia et al., 2018) |
|  | NEW | Secoxyloganin | (Jia et al., 2018) |
|  | LLF | Oleuropeinic acid | (Jia et al., 2018) |
|  | LLF | Neonuezhenide | (Jia et al., 2018) |
|  | LLF | Specnuezhenide | (Jia et al., 2018) |
|  | LLF | Nuezhenide | (Jia et al., 2018) |
|  | LLF | Oleuropein | (Jia et al., 2018) |
|  | LLF | Oleonuezhenide | (Jia et al., 2018) |
|  | LLF | 6’-O-trans-cinnamoyl-8-epikingisidic acid | (Jia et al., 2018) |
|  | LLF | 6'-O-cis-cinnamoyl-8-epikingisidic acid | (Jia et al., 2018) |
|  | LLF | 6’-elenolylnicotiflorine | (Jia et al., 2018) |
|  | LLF | Sibiricose A3 | (Zhu et al., 2023) |
|  | LLF | 8-demethyl-7-ketoliganin | (Zhu et al., 2023) |
|  | LLF | Ligustroside | (Zhu et al., 2023) |
|  | LLF | Ligustrosidic acid | (Zhu et al., 2023) |
|  | LLF | 10-hydroxyoleuropein or Ligustaloside A | (Zhu et al., 2023) |
|  | LLF | Acetylnicotiflorine | (Zhu et al., 2023) |
|  | LLF | Osmanthuside H | (Jia et al., 2018) |
|  | NEW | 8-O-acetylharpagide | (Jia et al., 2018) |
|  | LLF | D-valerolactone | (Zhu et al., 2023) |
| Triterpenoids | LLF; EH | Oleanolic acid | (Jia et al., 2018) |
|  | NEW | Eclalbasaponin X or Eclalbasaponin IX | (Jia et al., 2018) |
|  | EH | Ecliptasaponin Ⅵ | (Jia et al., 2018) |
|  | EH | Ecliptasaponin C or I or IV | (Zhu et al., 2023) |
|  | NEW | Ecliptasaponin IX | (Zhu et al., 2023) |
|  | EH | Ecliptasaponin V | (Jia et al., 2018) |
|  | EH | Ecliptasaponin A or D | (Jia et al., 2018) |
|  | EH | Ecliptasaponin A | (Zhu et al., 2023) |
|  | EH | Ecliptasaponin D | (Zhu et al., 2023) |
|  | EH | Eclalbasaponin Ⅴ | (Jia et al., 2018) |
|  | EH | Echinocystic acid | (Jia et al., 2018) |
|  | LLF | Betulin | (Zhu et al., 2023) |
|  | LLF | Tormentic acid | (Jia et al., 2018) |
|  | LLF | 3-O-cis-p-Coumaroyltormentic acid or  3-O-trans-p-Coumaroyltormentic acid | (Jia et al., 2018) |
|  | NEW | 16-hydroxy-3-oxoolean-12-en-28-oic acid | (Jia et al., 2018) |
|  | NEW | 3-hydroxy-11-ursen-28,13-olide | (Jia et al., 2018) |
|  | LLF | 3β-O-Acetylpomolic acid | (Jia et al., 2018) |
| Flavonoids | NEW | Skullcapflavone II | (Jia et al., 2018) |
|  | NEW | 4',7-dihydroxyl-3',6'-dimethoxylisoflavone-7-O-glucoside | (Jia et al., 2018) |
|  | LLF | Ligustroflavone | (Zhu et al., 2023) |
|  | LLF; EH | Quercetin | (Zhu et al., 2023) |
|  | LLF; EH | Apigenin | (Jia et al., 2018) |
|  | LLF; EH | Luteolin | (Jia et al., 2018) |
|  | EH | Luteolin sulfate | (Zhu et al., 2023) |
|  | LLF | Hyperoside | (Zhu et al., 2023) |
|  | EH | Isoquercitrin (Isoquercetin) | (Zhu et al., 2023) |
|  | LLF | Quercetin-3-O-rutinoside/rutin | (Zhu et al., 2023) |
|  | LLF | Luteolin-O-rutinoside | (Jia et al., 2018) |
|  | LLF; EH | Luteoloside (Luteolin-7-O-glucoside/luteolin-5-O-glucoside) | (Jia et al., 2018) |
|  | LLF; EH | Apigenin-7-O-glucoside | (Jia et al., 2018) |
|  | EH | Acacetin | (Jia et al., 2018) |
|  | EH | Acacetin-7-O-rutinoside | (Jia et al., 2018) |
|  | NEW | Kaempferol-3-sulfate | (Jia et al., 2018) |
|  | LLF | Daidzein | (Zhu et al., 2023) |
| Phenylethanols | LLF | Hydroxytyrosol | (Jia et al., 2018) |
|  | LLF | Salidroside | (Jia et al., 2018) |
|  | LLF | Echinacoside | (Jia et al., 2018) |
|  | LLF | Verbascoside | (Jia et al., 2018) |
|  | LLF | β-D-glucopyranoside-2-(4-hydroxyphenethyl)-6-acetate | (Zhu et al., 2023) |
|  | LLF | β-hydroxyverbascoside | (Zhu et al., 2023) |
| Benzoic acids | EH | 3,4-dihydroxybenzoic acid ethyl ester (Ethyl protocatechuate) | (Zhu et al., 2023) |
|  | NEW | 2,3-dihydroxybenzoic acid | (Zhu et al., 2023) |
| Anthraquinones | NEW | Emodin | (Zhu et al., 2023) |
|  | NEW | Emodin-3-methyl ether (Physcione) | (Zhu et al., 2023) |
| Phenolics | LLF | 1,2-dihydroxybenzene | (Zhu et al., 2023) |
|  | NEW | Hirsutanonol 5-O-glucoside | (Jia et al., 2018) |
| Phenylpropanoids  excluding Coumarins | LLF | Protocatechualdehyde | (Zhu et al., 2023) |
|  | LLF; EH | Caffeic acid | (Zhu et al., 2023) |
|  | LLF; EH | Protocatechuic acid | (Jia et al., 2018) |
|  | NEW | Ethyl caffeate (Caffeic acid ethyl ester) | (Jia et al., 2018) |
|  | NEW | Neochlorogenic acid | (Jia et al., 2018) |
|  | LLF; EH | Chlorogenic acid | (Jia et al., 2018) |
|  | NEW | Cryptochlorogenic acid  4-dicaffeoylquinic acid | (Jia et al., 2018) |
|  | NEW | Isochlorogenic acid A  3,5-Dicaffeoylquinic acid | (Zhu et al., 2023) |
|  | NEW | Isochlorogenic acid B  4,5-Dicaffeoylquinic acid | (Zhu et al., 2023) |
|  | NEW | Isochlorogenic acid C  3,4-Dicaffeoylquinic acid | (Jia et al., 2018) |
|  | NEW | 3-Coumaric acid | (Zhu et al., 2023) |
|  | NEW | Fraxiresinol 1-O-glucoside | (Jia et al., 2018) |
| Coumarins | EH | Demethylwedelolactone | (Zhu et al., 2023) |
|  | EH | Wedelolactone | (Jia et al., 2018) |
| Organic acids | NEW | 2-Isopropylmalic acid | (Zhu et al., 2023) |
|  | NEW | Malic acid | (Zhu et al., 2023) |
|  | NEW | Succinic acid | (Zhu et al., 2023) |
|  | NEW | Trans-3-Indoleacrylic acid  (3-Indolylacrylic acid) | (Zhu et al., 2023) |
|  | NEW | Citric acid | (Zhu et al., 2023) |
|  | LLF | Quinic acid | (Zhu et al., 2023) |
|  | NEW | D-Gluconic acid | (Zhu et al., 2023) |
|  | NEW | 2,2`,5`,2``-Terthiophene-5-carboxylic acid | (Zhu et al., 2023) |
| Esters | NEW | Myristyl sulfate | (Zhu et al., 2023) |
|  | NEW | Dodecyl sulfate | (Zhu et al., 2023) |
| Carbohydrates | NEW | Benzyl gentiobioside | (Jia et al., 2018) |
|  | LLF | Mannitol | (Zhu et al., 2023) |

Owing to methodological constraints, the determination of whether certain compounds qualify as isomers remains challenging.

**Reference**

JIA, L., FU, L., WANG, X., YANG, W., WANG, H., ZUO, T., ZHANG, C., HU, Y., GAO, X. & HAN, L. (2018). Systematic Profiling of the Multicomponents and Authentication of Erzhi Pill by UHPLC/Q-Orbitrap-MS Oriented Rapid Polarity-Switching Data-Dependent Acquisition and Selective Monitoring of the Chemical Markers Deduced from Fingerprint Analysis. *Molecules,* 23**,** 3143. doi:10.3390/molecules23123143

ZHU, T., CHEN, W., HAN, C., GAO, Z., LIU, E., GAO, X., FU, Z. & HAN, L. (2023). A comprehensive study on the chemical constituents and pharmacokinetics of Erzhi Formula and Jiawei Erzhi Formula based on targeted and untargeted LC-MS analysis. *Curr Drug Metab*. doi:10.2174/1389200224666230130093412
