# Supplementary material for: The modern scientific interpretation of ancient wisdom: a review of the phytochemistry and pharmacology of Erzhi Pill and its constituent botanical drugs
Source: Front Pharmacol. 2026 Apr 21;17:1797126. doi: 10.3389/fphar.2026.1797126 (PMC13139087; doi:10.3389/fphar.2026.1797126)
Supplement: Supplementary file 5 [file Table4.docx]

Table S4. Basic information of Erzhi Pill and its associated Chinese botanical formulas involved in clinical research

| Formula | Origin and Form | Name of Traditional Chinese Botanical drugs | Taxonomic Classifications | Ref. |
| --- | --- | --- | --- | --- |
| Modified Erzhi granules | Granules; produced by Huarun 39 medical Limited through Share Ltd. | *Fructus Ligustri Lucidi* | *Ligustrum lucidum* W.T.Aiton [*Oleaceae*; Ligustri Lucidi Fructus] | (Chen et al., 2018) |
|  |  | *Eclipta* | *Eclipta prostrata* (L.) L. [*Asteraceae*; Ecliptae Herba] |  |
|  |  | *Herba Cistanches* | *Cistanche deserticola* Y. C. Ma or *Cistanche tubulosa* (Schenk)Wight [*Orobanchaceae*; Cistanches Herba] |  |
|  |  | *Cynomorium Songaricum* | *Cynomorium songaricum* Rupr. [*Cynomoriaceae*; Cynomorii Herba] |  |
|  |  | *Cortex Phellodendri* | *Phellodendron chinense* Schneid. [*Rutaceae*; Phellodendri Chinensis Cortex] or *Phellodendron amurense* Rupr. [*Rutaceae*; Cortex Phellodendri Amurensis] |  |
| Dan Zhi Qing Re formula | Granules; produced by China Resources Sanjiu Medical & Pharmaceutical, Ltd. | *Salvia miltiorrhiae* Bge (Danshen) | *Salvia miltiorrhiza* Bge. [*Lamiaceae*; Salviae Miltiorrhizae Radix et Rhizoma] | (Fu et al., 2016) |
|  |  | *Anemarrhena asphodeloides* Bge (Zhimu) | *Anemarrhena asphodeloides* Bge. [*Liliaceae*; Anemarrhenae Rhizoma] |  |
|  |  | *Eucommia ulmoides* Oliv. (Duzhong) | *Eucommia ulmoides* Oliv. [*Eucommiaceae*; Eucommiae Cortex] |  |
|  |  | *Psoralea corylifolia* L (Buguzhi) | *Psoralea corylifolia* L. [*Fabaceae*; Psoraleae Fructus] |  |
| Erzhi formula | Granules; produced by China Resources Sanjiu Medical & Pharmaceutical, Ltd. | *Ligustrum Lucidum* Ait (Nvzhenzi) | *Ligustrum lucidum* W.T.Aiton [*Oleaceae*; Ligustri Lucidi Fructus] | (Fu et al., 2016) |
|  |  | *Eclipta prostrate* L (Mohanlian) | *Eclipta prostrata* (L.) L. [*Asteraceae*; Ecliptae Herba] |  |
| Er Zhi Tian Gui formula | Granules; produced by the Drug Manufacturing Unit of the Affiliated Hospital of Shandong University of Traditional Chinese Medicine; packaged as 3 g/bag; batch number 01-FZ032-03. | *Cuscuta chinensis* (Tu Si Zi) | *Cuscuta chinensis* Lam. [*Convolvulaceae*; Cuscutae Semen] | (Fang et al., 2013, Han et al., 2023, Liu et al., 2023, Sun et al., 2021) |
|  |  | *Ligustrum lucidum* (Nv Zhen Zi) | *Ligustrum lucidum* W.T.Aiton [*Oleaceae*; Ligustri Lucidi Fructus] |  |
|  |  | Herba Ecliptae (Mo Han Lian) | *Eclipta prostrata* (L.) L. [*Asteraceae*; Ecliptae Herba] |  |
|  |  | Fructus Lycii (Gou Qi Zi) | *Lycium barbarum* L. [*Solanaceae*; Lycii Fructus] |  |
|  |  | Angelica sinensis (Dang Gui) | *Angelica sinensis* (Oliv.) Diels [*Apiaceae*; Angelicae Sinensis Radix] |  |
|  |  | Radix Rehmanniae Preparata (Shu Di Huang) | *Rehmannia glutinosa* Libosch. [*Scrophulariaceae*; Rehmanniae Radix Praeparata] |  |
|  |  | Ligusticum wallichii (Chuan Xiong) | *Ligusticum chuanxiong* Hort. [*Apiaceae*; Chuanxiong Rhizoma] |  |
|  |  | Paeonia lactiflora (Bai Shao) | *Paeonia lactiflora* Pall. [*Ranunculaceae*; Paeoniae Radix Alba] |  |
|  |  | Rhizoma cyperi (Xiang Fu) | *Cyperus rotundus* L. [*Cyperaceae*; Cyperi Rhizoma] |  |
|  |  | Radix Glycyrrhizae Preparata (Zhi Gan Cao) | *Glycyrrhiza uralensis* Fisch., *Glycyrrhiza inflata* Bat., or *Glycyrrhiza glabra* L. [*Fabaceae*; Glycyrrhizae Radix Et Rhizoma Praeparata Cum Melle] |  |

Note: Name of Traditional Chinese Botanical drugs information was sourced from the original text. Taxonomic classification information was determined using the Kew Medicinal Plant Names Service (MPNS) website (http://mpns.kew.org/mpns-portal/).

**References**

Chen, R., Song, D., Zhang, W., Fan, G., Zhao, Y. & Gao, X. (2018). Randomized, Double-Blind, Placebo-Controlled Study of Modified Erzhi Granules in the Treatment of Menopause-Related Vulvovaginal Atrophy. *Evid Based Complement Alternat Med,* 2018**,** 6452709. doi:10.1155/2018/6452709

Fang, L., Rui-Xia, W., Feng-Mei, M., Zhen-Gao, S., Li-Hong, W. & Lei, S. (2013). Effects of Chinese medicines for tonifying the kidney on DNMT1 protein expression in endometrium of infertile women during implantation period. *J Altern Complement Med,* 19**,** 353-9. doi:10.1089/acm.2011.0410

Fu, S. F., Zhao, Y. Q., Ren, M., Zhang, J. H., Wang, Y. F., Han, L. F., Chang, Y. X., Fan, G. W., Wang, H., Huang, Y. H., Zhai, J. B., Dong, J. Y., Li, X., Ai, J. Q., Zhang, H., Zhu, Y., Zhang, B. L., Sun, L. K., Fan, X. & Gao, X. M. (2016). A randomized, double-blind, placebo-controlled trial of Chinese herbal medicine granules for the treatment of menopausal symptoms by stages. *Menopause,* 23**,** 311-23. doi:10.1097/gme.0000000000000534

Han, Q. S., Zhou, Y., Chen, W., Song, J. Y. & Sun, Z. G. (2023). Han, Q. S., Zhou, Y., Chen, W., Song, J. Y. & Sun, Z. G. (2023b). The role of Erzhi Tiangui formula in expected poor ovarian responders undergoing in vitro fertilization-embryo transfer: A multicenter, randomized, double-blind, placebo-controlled trial. *Medicine (Baltimore),* 102**,** e34088. doi:10.1097/md.0000000000034088

Liu, D. Q., Wei, C. F., Zhang, X., Xiang, S. & Lian, F. (2023). MicroRNA profiling reveals effects of Erzhi Tiangui granules on kidney deficiency diminished ovarian reserve: A randomized trial. *Medicine (Baltimore),* 102**,** e33652. doi:10.1097/md.0000000000033652

Sun, J., Song, J. Y., Dong, Y., Xiang, S. & Guo, Q. (2021). Erzhi Tiangui Granules Improve In Vitro Fertilization Outcomes in Infertile Women with Advanced Age. *Evid Based Complement Alternat Med*, 2021, 9951491. doi:10.1155/2021/9951491
